# Expression of lectin-like transcript-1 in human tissues

**DOI:** 10.12688/f1000research.10009.1

**Published:** 2016-12-29

**Authors:** Alba Llibre, Lucy Garner, Amy Partridge, Gordon J. Freeman, Paul Klenerman, Chris B. Willberg

**Affiliations:** 1 Peter Medawar Building for Pathogen Research, University of Oxford, Oxford, UK; 2 Department of Medical Oncology, Dana-Farber Cancer Institute, Harvard Medical School, Boston, USA; 3 Oxford NIHR Biomedical Research Centre, Oxford, UK

**Keywords:** Lectin-Like Transcript 1 (LLT1), C-type lectins, immune-privilege, human, distribution, natural killer cell

## Abstract

*Background:* Receptor-ligand pairs of C-type lectin-like proteins have been shown to play an important role in cross talk between lymphocytes, as well as in immune responses within concrete tissues and structures, such as the skin or the germinal centres. The CD161-Lectin-like Transcript 1 (LLT1) pair has gained particular attention in recent years, yet a detailed analysis of LLT1 distribution in human tissue is lacking. One reason for this is the limited availability and poor characterisation of anti-LLT1 antibodies. *Methods:* We assessed the staining capabilities of a novel anti-LLT1 antibody clone (2H7), both by immunohistochemistry and flow cytometry, showing its efficiency at LLT1 recognition in both settings. We then analysed LLT1 expression in a wide variety of human tissues. *Results:* We found LLT1 expression in circulating B cells and monocytes, but not in lung and liver-resident macrophages. We found strikingly high LLT1 expression in immune-privileged sites, such as the brain, placenta and testes, and confirmed the ability of LLT1 to inhibit NK cell function. *Conclusions:* Overall, this study contributes to the development of efficient tools for the study of LLT1. Moreover, its expression in different healthy human tissues and, particularly, in immune-privileged sites, establishes LLT1 as a good candidate as a regulator of immune responses.

## Background

Receptor-ligand pairs of C-type lectin-like proteins have been shown to play an important role in cross-talk between lymphocytes and in immune responses within tissues. Three examples have been well characterised in humans. These are the NKp65-Keratinocyte associated C type lectin (KACL), the NKp80-Activated induced C-type lectin (AICL) and the CD161-Lectin-Like Transcript 1 (LLT1), which are involved in skin immunobiology ^1^, cross-talk between Natural Killer (NK) cells and monocytes ^2^ and modulation of T, NK and B cell immune responses ^3– 6^, respectively. Amongst these, the CD161-LLT1 pair has been the focus of attention of several recent studies ^6– 10^. LLT1 has been described as a multi-functional protein ^11^, and to fully elucidate the functional consequences of its interactions with its receptor, CD161, a comprehensive characterisation of LLT1 distribution is needed. The current published literature presents inconsistencies, which may partially be due to the activation state of the cells tested and the different anti-LLT1 antibodies used. Indeed, LLT1 has been shown to be upregulated upon different forms of activation ^6, 12– 15^.

Tissues within the body display varying antigenic profiles, and the expression of specific molecules is involved in the maintenance of tissue function. Tissue grafts placed in particular anatomical structures can avoid rejection for long periods of time ^16^. This observation led to the notion of immune-privilege, believed to be an evolutionary adaptation to protect essential organs from harmful inflammatory responses. At first, it was thought that antigens did not have access to immune-privileged sites, thus avoiding a response. However, more recent evidence suggested that the maintenance of immune-privilege relies on active rather than passive mechanisms ^17, 18^. Some examples include: a lack of lymphatic drainage, low expression of MHC class I molecules, local production of immunosuppressive cytokines, as well as enhanced expression of inhibitory surface molecules ^19^. Immunologically privileged sites include the brain, the eyes, the placenta, the fetus and the testes. Although there has been abundant research regarding the mechanisms behind effective suppression of inflammatory responses in immune-privileged structures, further studies are required to fully elucidate and understand them ^20^.

The main aim of this study was to broadly characterise the expression of LLT1 within the human body. We screened a wide variety of human cell types and tissues using our novel monoclonal antibody, clone 2H7, and described LLT1 expression in circulating B cells and monocytes. The presence of LLT1 could also be observed in B cells in tonsils, as previously described ^6, 9, 21^, but not in Kupffer cells in the liver or alveolar macrophages in the lung. Furthermore, LLT1 could be detected in several healthy human tissues, but it was remarkably prevalent in immune-privileged sites, such as brain, placenta and testes. We also confirmed the previously described phenomenon that LLT1 inhibits NK cell function ^4, 5, 14, 22^.

Overall, the current study contributes to the development of effective tools for the study of LLT1. We characterised the strong expression of this C-type lectin in B cells, monocytes and immune-privileged tissues; thus, postulating a role for LLT1 in cross talk between lymphocytes and immune tolerance.

## Materials and methods

### Cell lines

The 300.19 cell line is an Abelson leukemia virus transformed murine pre-B cell line derived from Swiss Webster mice (H-2 ^d^). They were maintained in RPMI 1640 (Sigma-Aldrich) supplemented with 10% fetal calf serum (FCS; PAA Laboratories), 1% streptomycin/penicillin (Sigma Aldrich), 1% L-glutamine (Sigma Aldrich), 15mg/ml gentamycin (Sigma Aldrich) and 50 × 10 ^-6^ M β-mercaptoethanol (Sigma Aldrich).

The cell lines 300.19-CD161 and 300.19-LLT1 are 300.19 cells transfected with a vector expressing human CD161/LLT1 cDNA and the puromycin resistance gene. These cell lines were maintained in the same media as 300.19 cells, with the addition of puromycin (5mg/ml; Gibco Life Technologies). All three 300.19 cell lines were kept at 37°C, 5% CO _2_ and split 1:10 three times a week.

All three 300.19 cell lines were kindly gifted by Gordon Freeman (Harvard Medical School, Boston, MA, USA).

### Tissues

A series of normal paraffin-embedded human tissues comprising samples of tonsil, liver and lung were obtained from Proteogenix.

Tonsils were also obtained following routine tonsillectomy from the ENT Department at the John Radcliffe Hospital, Oxford. Ethical approval was obtained from the John Radcliffe Hospital, and written informed consent was obtained from all subjects.

Formalin-fixed, paraffin-embedded healthy and tumour tissue arrays were obtained from AMS Biotechnology.

### Primary cells and B cell purification

Peripheral Blood Monocluear cells (PBMCs), obtained from the National Blood Transfusion Service, were isolated on a Lymphoprep gradient (Axis Shield), aliquoted in FCS + 10% dimethyl sulfoxide (Sigma Aldrich) and stored in liquid nitrogen until required.

B cells were isolated by negative magnetic selection using EasySep™ Human B cell Enrichment Kit (STEMCELL technologies), following the manufacturer’s instructions.

### Novel mouse anti-human LLT1 monoclonal antibody

Clone, 359. **2H7**; mIgG2a; kappa; dilution, 1.42 mg/ml. Validated by flow cytometric stain of human LLT1 transfected cells. The purified antibody is dialysed against phosphate buffered saline (PBS), is low in endotoxin (< 2EU/mg), and is sterile filtered. This antibody was generated in the laboratory of Gordon Freeman, Harvard Medical School (Boston, MA, USA). For immunohistochemical and flow cytometry stainings, the 2H7 antibody was used at 1:500 and 1:50 dilution, respectively, in PBS.

### Fluorescence-activated cell sorting (FACS)

For external staining, cells from cell lines or PBMCs in PBS were incubated with anti-surface antibodies at room temperature (RT) for 20 min. Live/dead staining was performed using LIVE/DEAD® Fixable Near-IR Dead Cell Stain Kit(Invitrogen), at 633 or 635 nm excitation.

For internal staining, cells were fixed with 2% formaldehyde (Sigma Aldrich) in PBS for 10 min and permeabilized with IX permeabilization buffer (eBioscience) in water.

The following antibodies were used: CD3-FITC (BioLegend, Catalog No. 300406, clone UCHT1, Mouse IgG1, k), CD8-PerCP-Cy5.5 (BioLegend, Catalog No. 344710, clone SK1, Mouse IgG1, k), CD38-PerCP-Cy5.5 (BioLegend, Catalog No. 303522, clone HIT2, Mouse IgG1, k), CD56-APC (Biolegend, Catalog No. 318310, clone HCD56, Mouse IgG1, k); CD19-BV421 (BD Bioscience, Catalog No. 562441, clone HIB19, mouse IgG1, k); CD4-VioGreen (Miltenyi Biotec, Catalog No. 130-106-712, clone M-T466, Mouse IgG1, k), CD161-PE (Miltenyi Biotec, Catalog No. 130-092-677, clone 191B8, Mouse IgG2a), IgG2A isotype control (R&D Systems, Catalog No. MAB003, mouse); and 2H7 mAb. When non-conjugated primary antibodies were used, a secondary rat anti-mouse IgG2A-PE (R&D Systems, Catalog No. F0129, clone 344701, IgG1) was used.

FACS analysis was performed on Miltenyi Biotec MACSQuant cytometer and analyzed with FlowJo Version 9.6.2 software (TreeStar).

### Immunohistochemistry

Tissue deparaffinisation was performed using Histo-Clear (National Diagnostics) and ethanol (Sigma Aldrich; 100%, 90% and 70%). Heat mediated antigen retrieval was achieved using Dako target retrieval solution (Dako). Endogenous peroxidase activity was blocked using 3% H _2_O _2_ (5 min × 2; Alfa Aesar) and 0.1% sodium azide (15 min; Sigma Aldrich) in water. Non-specific binding was blocked by incubating the sample for 30 min at RT with 0.5% blocking reagent (PerkinElmer) in PBS. The 2H7 mAb or IgG2A isotype control (R&D Systems) (3 μg/ml) were added and incubated overnight at 4°C. The sample was then incubated with horse anti-mouse polymer horseradish peroxidase (HRP)-conjugated (Vector Laboratories, Catalog No. MP-7402) for 30 min at RT. ImmPACT DAB peroxidase substrate (Vector Laboratories) was added and incubated for 2–10 min. The reaction was stopped with running deionised water. The section was covered with hematoxylin (Vector Laboratories) for 45 seconds and rinsed with deionised water. Samples were then dehydrated by serial passage through 70%, 90% and 100% ethanol followed by Histo-Clear. Samples were allowed to dry and mounted with VectaMount mounting media (Vector Laboratories). For analysis of immunohistochemical staining, images were acquired on a DSS1 Coolscope Slide Scanner (Nikon).

For immunofluorescent staining, the following primary antibodies were used: anti-LLT1 (R&D Systems, Catalog No. AF3480, goat polyclonal) and anti-CD68 (DAKO, Catalog No. M0876, clone PG-M1, mouse IgG3, k). They were diluted in blocking buffer and incubated for 30 min at RT. Anti-goat HRP-conjugated polymer was added followed by a 30 min incubation at RT. Cyanine 5 Amplification Reagent (PerkinElmer) was diluted 1/300 in Tyramide amplification buffer [12ml 500mM Tris, 18ml H _2_O, 20 mg Imidazole (Sigma Aldrich), O _2_], added and incubated in the dark for 15 min. Residual peroxidase activity was blocked by incubating the slides in 3% H _2_O _2_ for 5 min and then 0.1% sodium azide for 15 min in water. Anti-mouse HRP-conjugated antibody was added followed by a 30 min incubation at RT. Fluorescein Amplification Reagent (PerkinElmer) was diluted 1/300 in new Tyramide amplification buffer, added and incubated in the dark for 15 min. Slides were mounted with ProLong® Gold Antifade Reagent with DAPI (Invitrogen). For immunofluorescent microscopy, images were acquired on an Olympus Fluoview FV1000 microscope (Olympus) and analyzed using Fiji (ImageJ v1.47h); National Institute of Health, USA).

### NK cell degranulation assay

In total, 2 × 10 ^5 ^PBMCs per well were seeded in 100μl R10 with IL-15 and IL-2 (1 ng/ml each; PeproTech) and incubated over night at 37°C with 5% CO _2_. A total of 4 × 10 ^4^ of 300.19 or 300.19-LLT1 cells were added, together with the CD107a-PE-Cy7 (1/1000; BioLegend) and incubated for 1h at 37°C. Monensin (1/1000; BioLegend) was added and the cells were incubated for another 4–5h before the cells were stained for FACS.

### Data analysis

Graphs and statistical analysis were performed using GraphPad Prism Version 6.0a (GraphPad Software) and Adobe Illustrator CS4 14.0.0.

## Results

### LLT1 is expressed on tonsillar B cells and circulating B cells and monocytes, but not on lung and liver-resident macrophages

There are still many inconsistences in the published data regarding the distribution of LLT1 in human tissues and cell types. Some past studies reported LLT1 expression in resting PBMCs, whereas others could only detect it after activation ^13– 15^.

**Figure 1.**
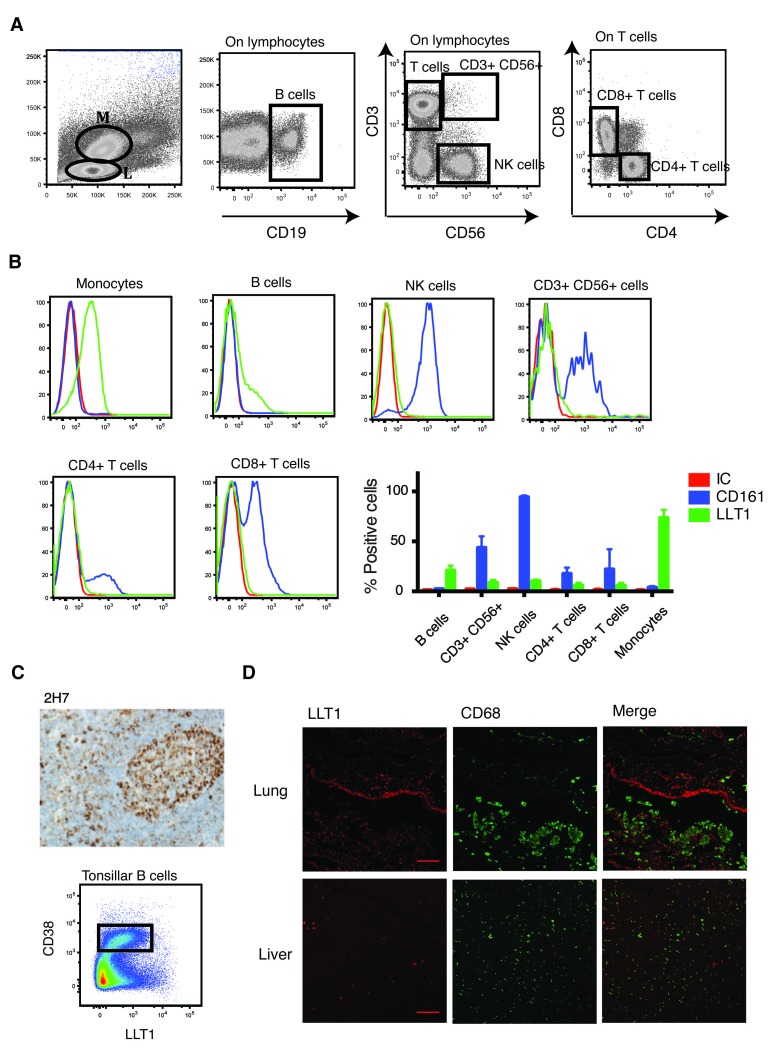
LLT1 is expressed on B cells and monocytes. Lectin-like transcript 1 (LLT1) (stained using the 2H7 mAb) and CD161 levels were measured by flow cytometry in monocytes (M) and lymphocytes (L). ( **A**) The gating strategy, ( **B**) representative and cumulative data for the expression of CD161 (blue) and LLT1 (green) compared to the isotype control (IC, red) (n=4). ( **C**) Representative image of LLT1 staining in human tonsil tissue using the 2H7 anti-LLT1 antibody (10×; n=6), together with a representative FACS plot of LLT1 staining of purified tonsillar B cells with the 2H7 antibody. The GC population is highlighted (rectangle; n=8). ( **D**) Immunofluorescent co-staining of LLT1 (red) and CD68 (green) in lung and liver (scale bar = 100μm). Representative of two independent experiments.

We assessed the presence of this C-type lectin in resting and activated PBMCs. We analysed different cell subsets ( Figure 1A) and detected abundant expression of LLT1 in resting monocytes (60–80%) and B cells (15–30%) ( Figure 1B). These results fit with the previously characterised expression of LLT1 in B-cell derived Raji cells ^5, 13, 23^ and monocyte-derived THP-1s ^23^. Interestingly, the receptor ligand pair LLT1-CD161 was expressed on PBMCs in an exclusive manner ( Figure 1B). While monocytes and B cells expressed LLT1, their levels of CD161 expression were null. On the contrary, all the other subsets tested expressed CD161 to a certain extent, although they did not express its ligand, LLT1.

We next studied LLT1 presence in tissue-resident B cells and macrophages. We and others have shown the expression of LLT1 in tissue resident germinal centre B cells ^6, 9^. We demonstrated that the 2H7 mAb recognises LLT1 on germinal centre B cells, both immunohistologically and by flow cytometry ( Figure 1C) ^6^. Thus, the 2H7 mAb is a good tool for studying the distribution of LLT1 in tissue through immunohistochemical staining. Expression of this C-type lectin on tissue-resident macrophages had previously only been addressed in the joints of rheumatoid arthritis (RA) patients, which were positive for LLT1 ^10^. We wanted to assess the expression of LLT1 in macrophages resident in other tissues. In order to do so, we performed immunofluorescent staining of lung and liver sections, using LLT1 and the macrophage marker CD68. CD68 expressing alveolar macrophages could be detected in the lung, as well as CD68 expressing Kupffer cells in the liver. However, both cell types were negative for LLT1 ( Figure 1D; Dataset 1 ^34^), suggesting that terminally differentiated macrophages do not express this C-type lectin. Nonetheless, LLT1+ cells could be detected in both tissues, suggesting that these LLT1+ cells may, for example, be epithelial cells, but further work is needed for their full characterisation.

### LLT1 on activated PBMCs

**Figure 2.**
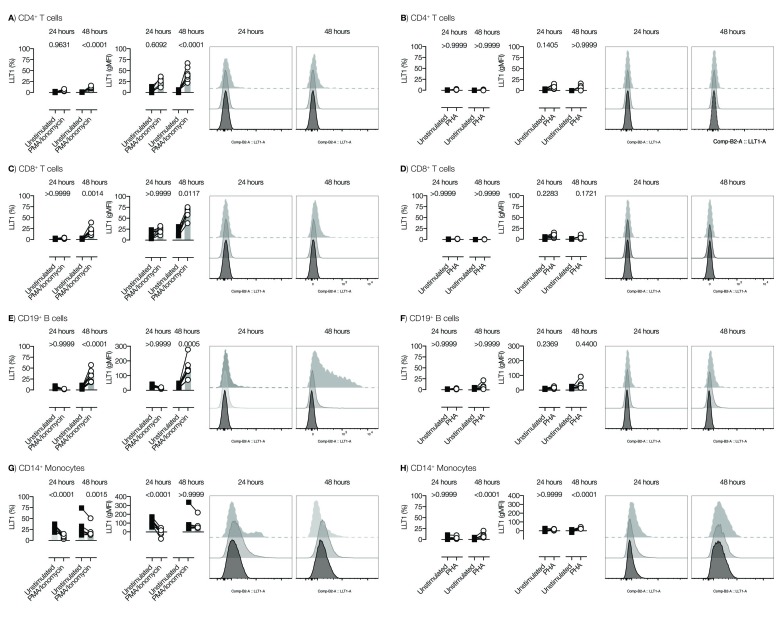
LLT1 on activated PBMCs. PBMCs were stimulated for either 24 or 48h with PMA/ionomycin or PHA. Lectin-like transcript 1 (LLT1) expression on different cell subsets was measured by FACS, and presented as the percentage of LLT1+ cells (left hand graphs) or the gMFI of LLT1 expression (middle graphs) within the given populations. Representative histograms showing isotype control (after 48h; light grey), or LLT1 expression at 24h (dark grey) and 48h (black) are shown in the right hand plots. Two-way ANOVA with Bonferroni’s multiple-comparisons test were applied (**<0.01, ***<0.001 , ****<0.0001). Data from two pooled experiments.

The expression of LLT1 on activated PBMCs was assessed. Stimulation with PMA/ionomycin for 24 and 48h had no significant effect on CD4+ T cells ( Figure 2A). Minimal levels of LLT1 were observed on CD8+ T cells after stimulation, although this did not reach significance ( Figure 2C). However, the expression levels were very low and of questionable biological relevance. Similar results were seen using PHA ( Figure 2B and D).

Interestingly, the percentage and levels of LLT1 on B cells initially dropped after 24h, but increased after 48h ( Figure 2E) upon stimulation with PMA/ionomocyin; a similar trend was observed with PHA ( Figure 2F).

Monocytes also lowered LLT1 expression upon activation after both 24 and 48h stimulation with PMA/ionomycin ( Figure 2G), but not after PHA stimulation ( Figure 2H).

### LLT1 is expressed in healthy human tissue and some tumours

**Figure 3.**
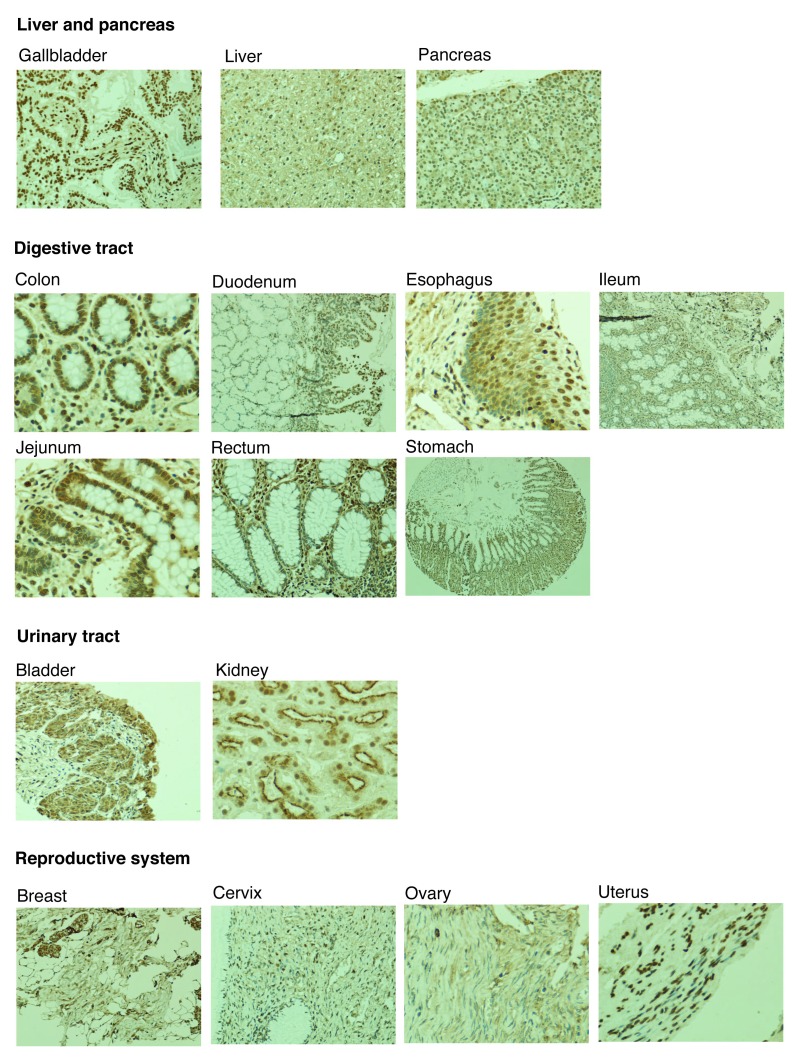
Expression of lectin-like transcript 1 (LLT1) in healthy immune tissue. Staining of human healthy tissue with the 2H7 anti-LLT1 antibody at 1/500. Representative images from three independent experiments. 5×, 10× and 20× magnification.

There have been limited attempts to characterise the distribution of LLT1 within human tissues. In this study, we screened a wide variety of human healthy tissues using the 2H7 antibody clone. A representative stain of each tissue tested is shown in Figure 3. LLT1 could be detected in a wide variety of tissues, such as the gallbladder and the digestive tract (glandular cells), as well as in the kidneys (cells in tubules) or the lung (pneumocytes). We also compared the expression pattern of LLT1 in healthy and tumour human tissues ( Supplementary Figure 1). Although LLT1 upregulation has been shown in glioblastoma and prostate cancer ^22, 24^, our results did not support this being a common trend in all cancerous tissues. Most likely, changes in LLT1 expression upon malignant transformation are tissue-dependent.

### LLT1 is expressed in immune-privileged sites

**Figure 4.**
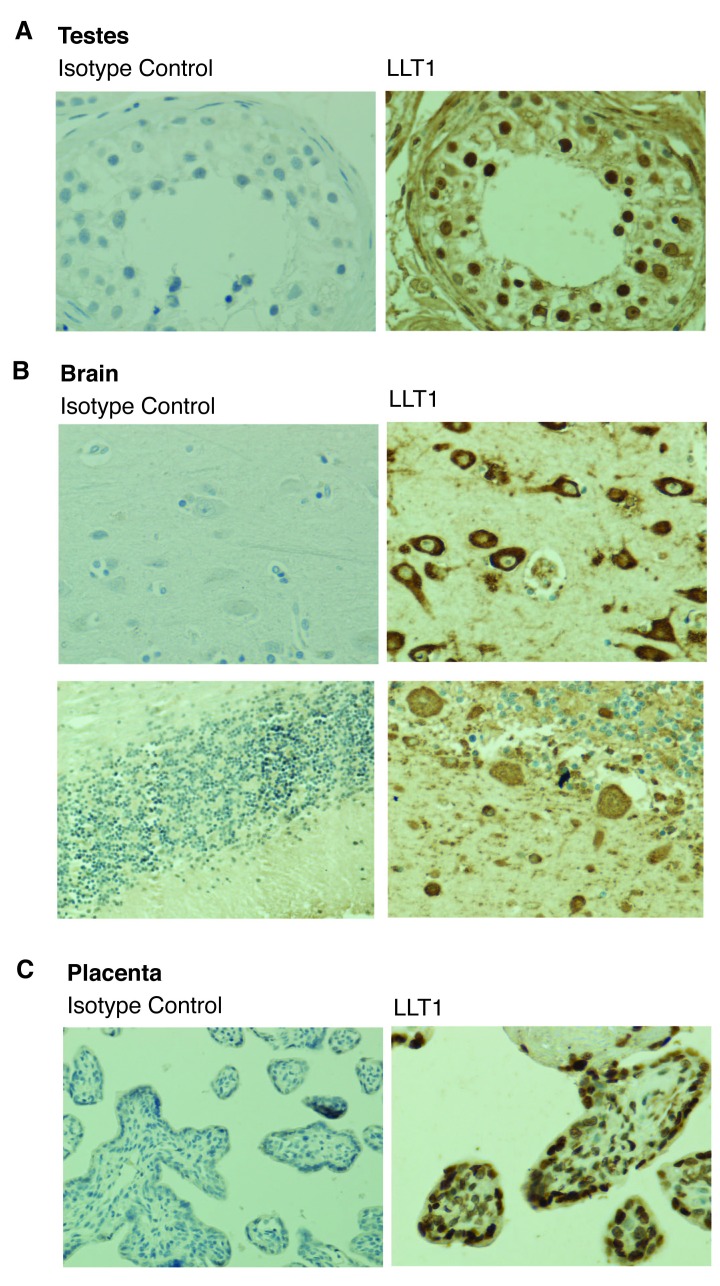
LLT1 is highly expressed in immune-privileged sites. Lectin-like transcript 1 (LLT1) and isotype control stainings of testes ( **A**), brain ( **B**) and placenta ( **C**) using the anti-LLT1 2H7 mAb (1/500). Representative image of three independent experiments. 20× magnification.

Although LLT1 could be detected in different human tissues ( Figure 3), its expression was strikingly high in immune-privileged sites ( Figure 4). Cells in the seminiferous ducts within the testes, trophoblastic cells in the placenta and neurons strongly expressed LLT1. Purkinje cells, a large type of neuron that resides in the cerebellum and release the neurotransmitter gamma-aminobutyric (GABA) was also found to be positive for LLT1. A key feature of immune privilege is low expression of MHC class I molecules, which protects certain tissues from excessive and damaging inflammatory T cell responses ^19^. However, downregulation of MHC class I molecules results in increased susceptibility to NK cell killing. Therefore, we next tested the effect of LLT1 on NK cell cytotoxic effector functions.

### LLT1 inhibits NK cell degranulation

**Figure 5.**
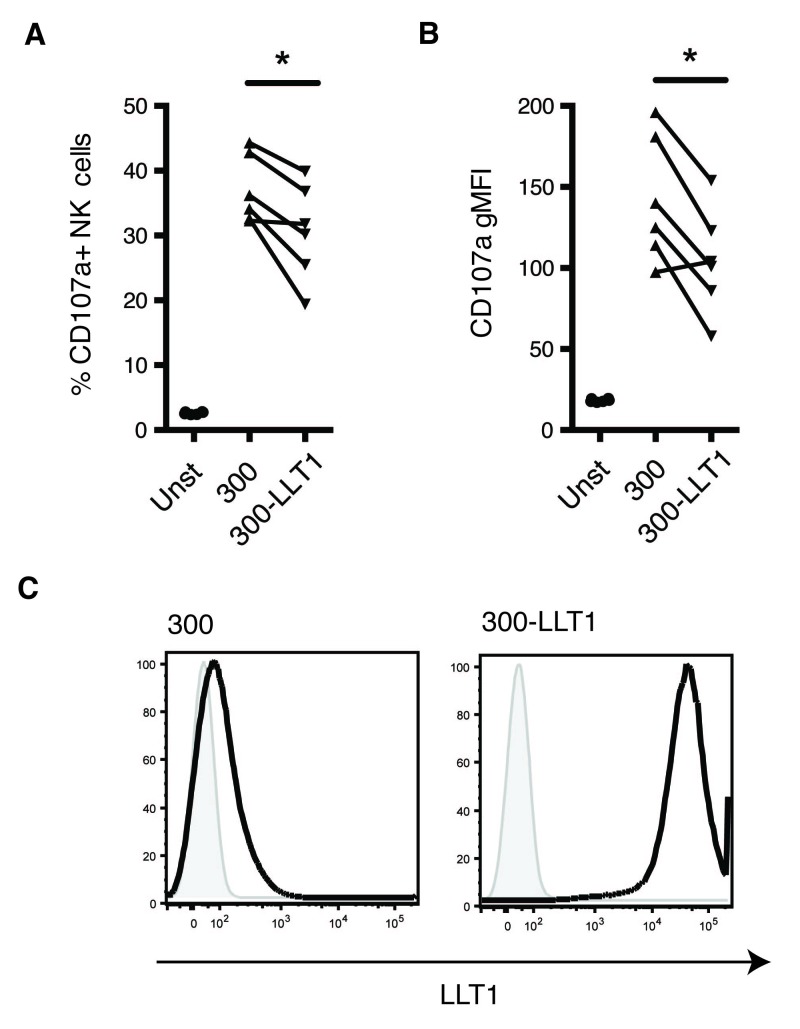
LLT1 inhibits NK cell degranulation. The percentage of CD107a- expressing NK cells, (gated on live, CD3- CD56+ cells) ( **A**), as well as CD107a geoMFI values ( **B**) with 300.19- lectin-like transcript 1 (LLT1) as targets compared to the untransfected 300.19 cells (*<0.05, non-parametric paired T-test; CD107a geoMFI: p value=0.0239; percentage of CD107a-expressing cells: p value=0.0239). Data pooled together from two independent experiments (n=6). ( **C**) Expression of LLT1 on untransfected 300.19 and 300.19-LLT1 cell lines. Representative histogram of three independent FACS stainings with the 2H7 anti-LLT1 antibody.

A role for LLT1 in suppression of NK cell function has been described ^4, 5, 14, 22^. Here, we confirmed that the presence of LLT1 reduces NK cell degranulation. NK cell surface expression of CD107a was reduced when NK cells were cultured with target cells, the 300.19 cell line transfected with LLT1, as compared to controls ( Figure 5A and B). Figure 5C shows expression levels of LLT1 on target cells, confirming very high levels of this C-type lectin in the transfected 300.19-LLT1 cells, as expected. In summary, our data suggests a plausible role for LLT1 in immune-regulation and, particularly, in negative modulation of NK cell responses in immune-privileged sites.

All staining and flow cytometry experiments undertaken by the present study Click here for additional data file. Copyright: © 2016 Llibre A et al. 2016 Data associated with the article are available under the terms of the Creative Commons Zero "No rights reserved" data waiver (CC0 1.0 Public domain dedication).

## Discussion

In humans, there are three well-characterised NKC-encoded receptor-ligand pairs: these are the CD161-LLT1, NKp65-KACL and NKp80-AICL. The expression of CD161 has been widely studied. It has been described on the vast majority of NK cells ^25^, on different innate-like T cell subset,s such as NKT cells ^3^, mucosal-associated invariant T (MAIT) cells ^26^, γδ T cells ^27, 28^ and in other T cell subgroups, both in the CD4+ and CD8+ compartments. CD161 defines cell populations with shared transcriptional and functional features across different human T cell lineages ^8^. In contrast, the expression and localisation of LLT1 has been much less studied.

LLT1 was first described on NK, T and B cells ^29^, although some subsequent studies showed different results ^12, 13^. We showed LLT1 expression on circulating B cells and monocytes, confirming the results obtained in previous research ^12^. It is important to note that the current literature still presents inconsistencies regarding LLT1 distribution in PBMCs, which could be due to the use of different antibodies as well as the diverse activation state of the cells tested ^5, 6, 12, 13^.

LLT1 has been shown in joint-resident macrophages of RA patients ^10^; however, we could not detect LLT1 on macrophages from the liver or the lung. These results could be explained by the state of activation of macrophages, suggesting an increase of LLT1 expression under inflammatory conditions. PMA/ionomycin and mitogen (PHA) stimulation of PBMCs demonstrated that a broad range of cell types could, to a limited degree, express some LLT1, which was dependent on the duration of the stimuli. In particular, B cells showed a bi-phasic expression pattern.

We showed expression of LLT1 in different healthy human tissues ( Figure 3) and, particularly, in immune-privileged sites ( Figure 4). It is believed that immune-privilege is the result of an evolutionary process that confers special immune tolerance to certain structures ^19^. Organs, such as the eye, the brain or the placenta, present the exceptional capacity of preventing classical inflammatory responses that could be highly detrimental or even fatal. This singular immune status is linked to low expression levels of MHC class I molecules, which subsequently lead to increased susceptibility to killing by NK cells. We and others have shown that the presence of LLT1 results in decreased NK cell function ( Figure 5) ^4, 5, 14^. Therefore, LLT1 could play a prominent role in keeping NK cells under control in immune-privilege sites, thus preventing damage of low-expressing MHC class I tissues. This hypothesis matches with the role described for the murine version of LLT1, mOCIL. The distribution of mOCIL differs substantially from its human homolog, as it is believed to be expressed almost ubiquitously, similarly to MHC class I molecules ^30, 31^.

A high degree of homology has been described for the mouse and human forms of the CLEC2D protein ^32^, suggesting that these antibodies could be reacting with both mouse (Clr-b) and human (LLT1) forms. The distribution of Clr-b has been widely studied, in contrast to the human one. However, so far, there is no particular mention of the presence of Clr-b in mouse B cells, although it has been described in nearly all haematopoietic cells and abundant mouse tissues, with some exceptions (i.e. the brain) ^30, 31^.

We found LLT1 and CD161 to be expressed in different subgroups of lymphocytes as well as monocytes ( Figure 1). We also described expression of LLT1 in various human healthy tissues and particularly in immune-privileged sites ( Figure 3 and Figure 4). It is tempting to speculate that this pair of C-type lectins is involved in the cross-talk between distinct LLT1 and CD161 expressing immune cell types, such as B cells and T cells or monocytes and NK cells. The closely related pair of C-type lectins NKp80-AICL follows this same pattern: they are expressed on NK cells and monocytes, respectively, playing a role in reciprocal cell activation ^2^. The other well-described human C-type lectin pair is NKp65-KACL, which is expressed mainly on NK cells and keratinocytes, respectively ^33^. Thus, this particular pair illustrates a very different case, as it is involved in the immune surveillance of a specific tissue (i.e. the skin). This framework could also apply to LLT1 and CD161, both in terms of lymphocyte/monocyte interaction and interaction between NK cells and immune-privileged sites, although these hypotheses require further investigation.

Overall, we have contributed to the development and optimisation of tools necessary for the study of LLT1. Its striking expression in immune-privileged sites, as well as its presence in different immune cell types establishes LLT1 as an excellent candidate for immune-regulation. A detailed understanding of LLT1 distribution, regulation and function will give great insights into our knowledge on how immune-privilege works, as well as helping us to comprehend tissue-specific immune responses during inflammation.

## Data availability

The data referenced by this article are under copyright with the following copyright statement: Copyright: © 2016 Llibre A et al.

Data associated with the article are available under the terms of the Creative Commons Zero "No rights reserved" data waiver (CC0 1.0 Public domain dedication). 

**Dataset 1: All staining and flow cytometry experiments undertaken by the present study.** Doi, 10.5256/f1000research.10009.d147459 ^34^.

## Supplementary material

**Supplementary Figure 1. LLT1 staining of human tumor tissues.** Staining of cancerous human tissues was performed using the 2H7 antibody (1/500). Changes in Lectin-Like Transcript 1 (LLT1) expression were tissue dependent. Examples of brain ( **A**), colon ( **B**) and uterus tissue are shown. 10× and 20× magnification.
