# Supplementary material for: Expression of lectin-like transcript-1 in human tissues
Source: F1000Res. 2016 Dec 29;5:2929. [Version 1] doi: 10.12688/f1000research.10009.1 (PMC5365220; doi:10.12688/f1000research.10009.1)
Supplement: All staining and flow cytometry experiments undertaken by the present study [file f1000research-5-10785-s0000.tgz › a5f7a3a6-96bf-48b5-9aa2-5312f4521761_Dataset_1.pdf]

Dataset 1

Raw data for Figure 1B. LLT1 and CD161 on PBMCs. Flow cytometric staining of monocytes and PBMCs (B cells, NK cells, CD3+ CD56+ cells, CD4+ T cells and CD8+ T cells) for the C-type lectins LLT1 and CD161.

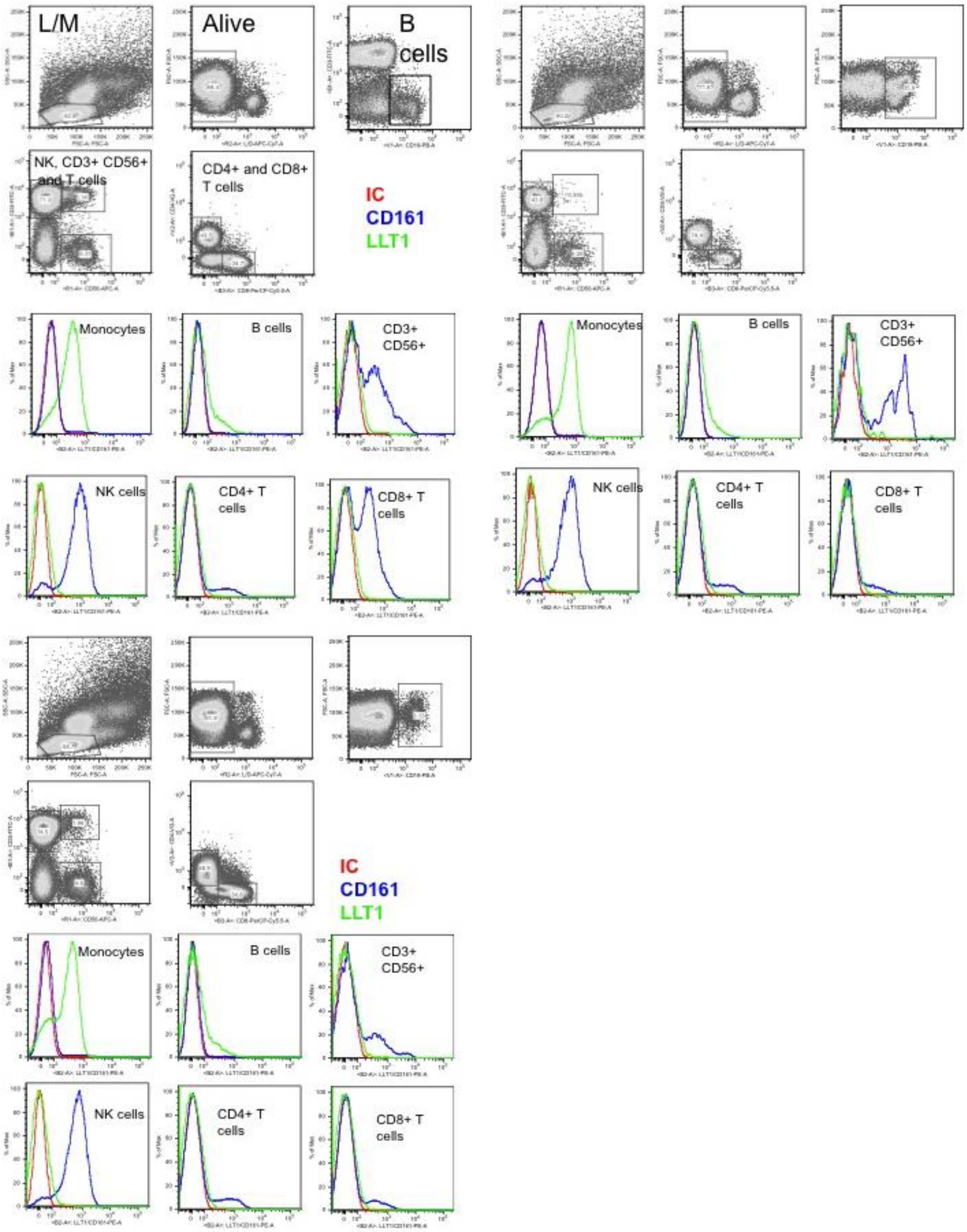

Raw data for Figure 1C. LLT1 staining of human tonsil. LLT1 staining in human tonsil tissue using the 2H7 anti-LLT1 antibody (5x, 10x and 20x), together with LLT1 FACS staining of purified tonsillar B cells with the 2H7 antibody.

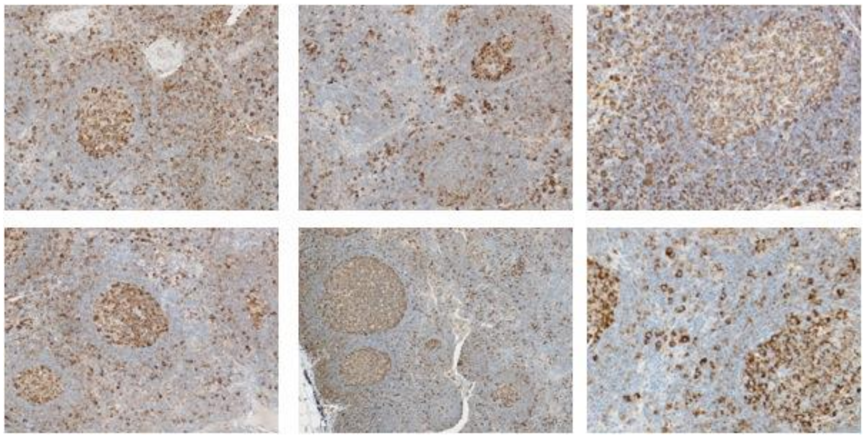

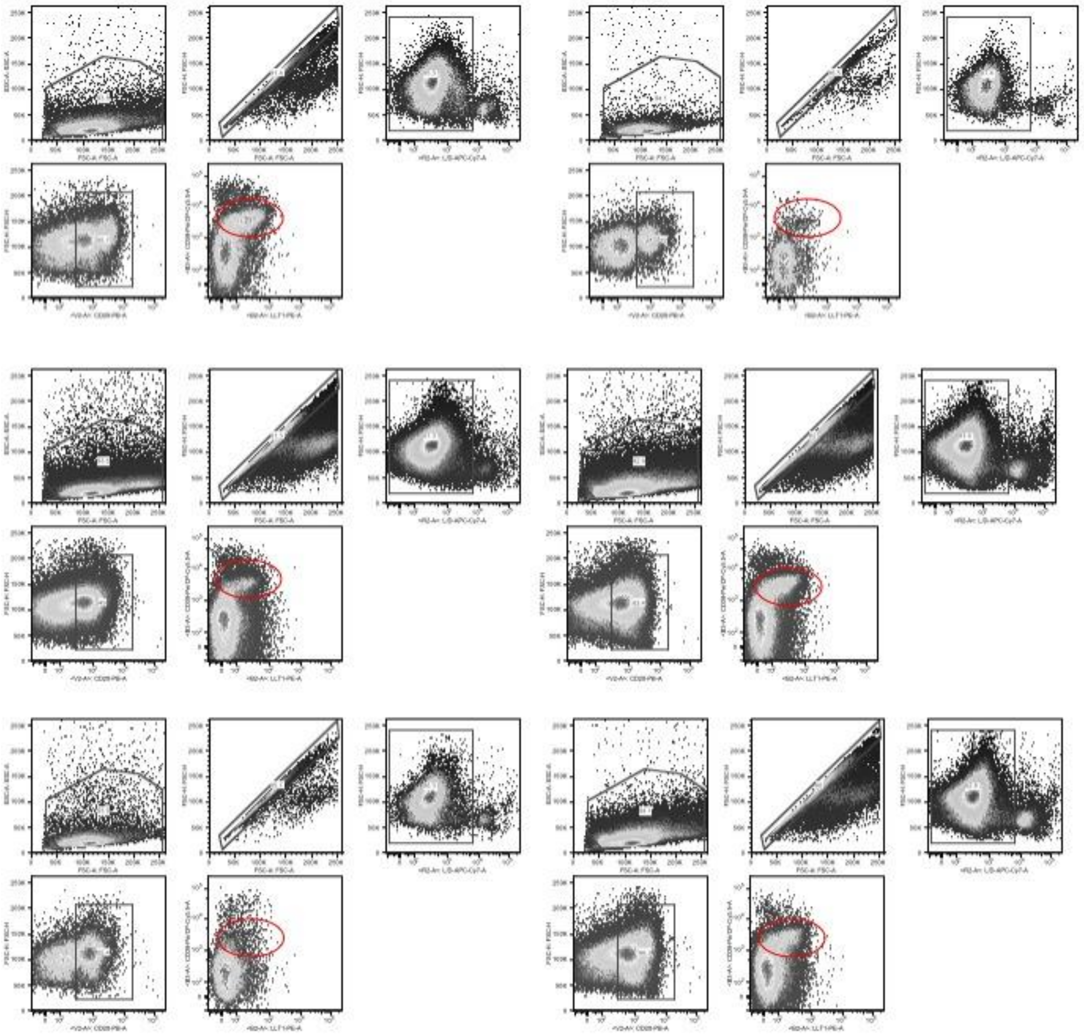

LLT1 is not expressed in liver and lung-resident macrophages.

Immunofluorescent staining of LLT1 (red) and CD68 (green) in lung and liver (scale bar = 100 μm) (Figure 1).

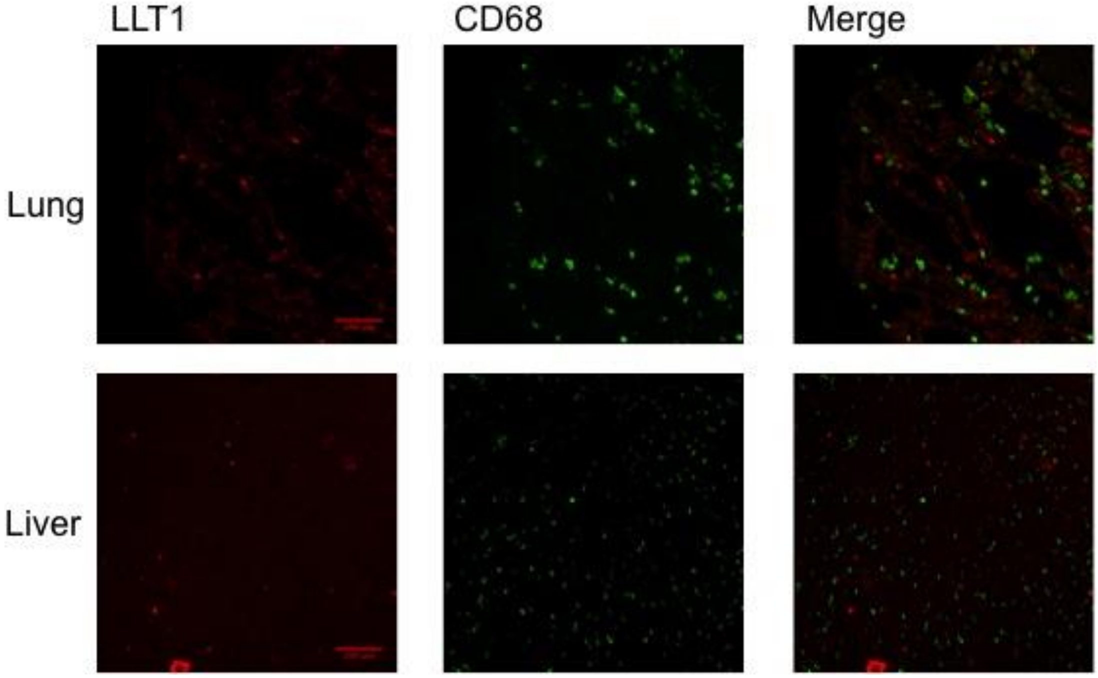

Raw data for Figure 2. LLT1 in activated PBMCs. Gating strategy and plots from individuals used to generate the results shown in Figure 2.

PMA/Ionomycin stimulation at 24h

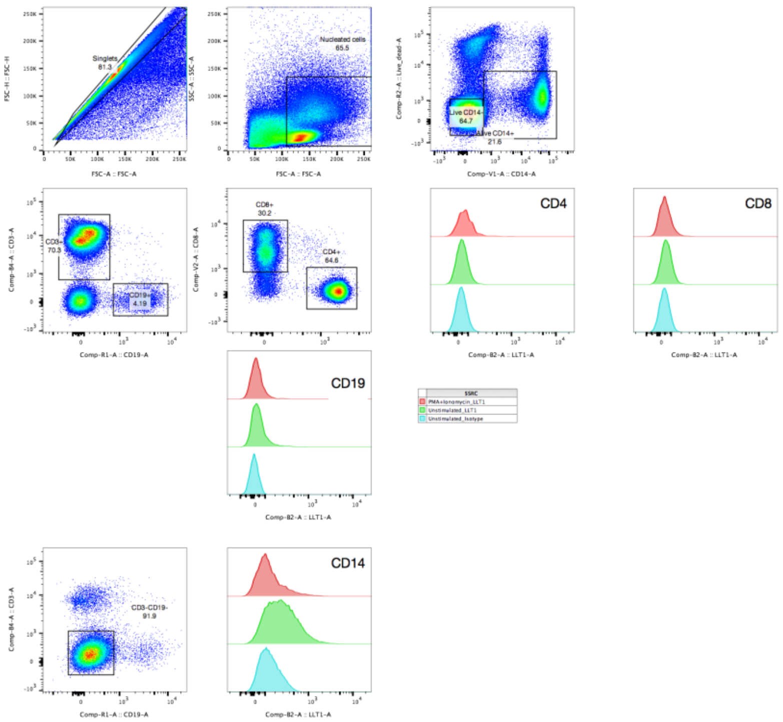

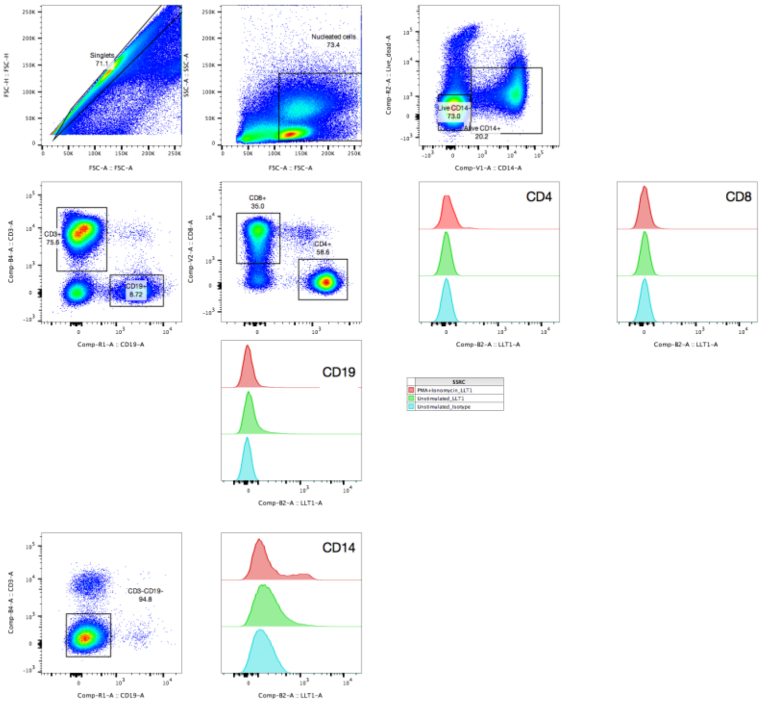

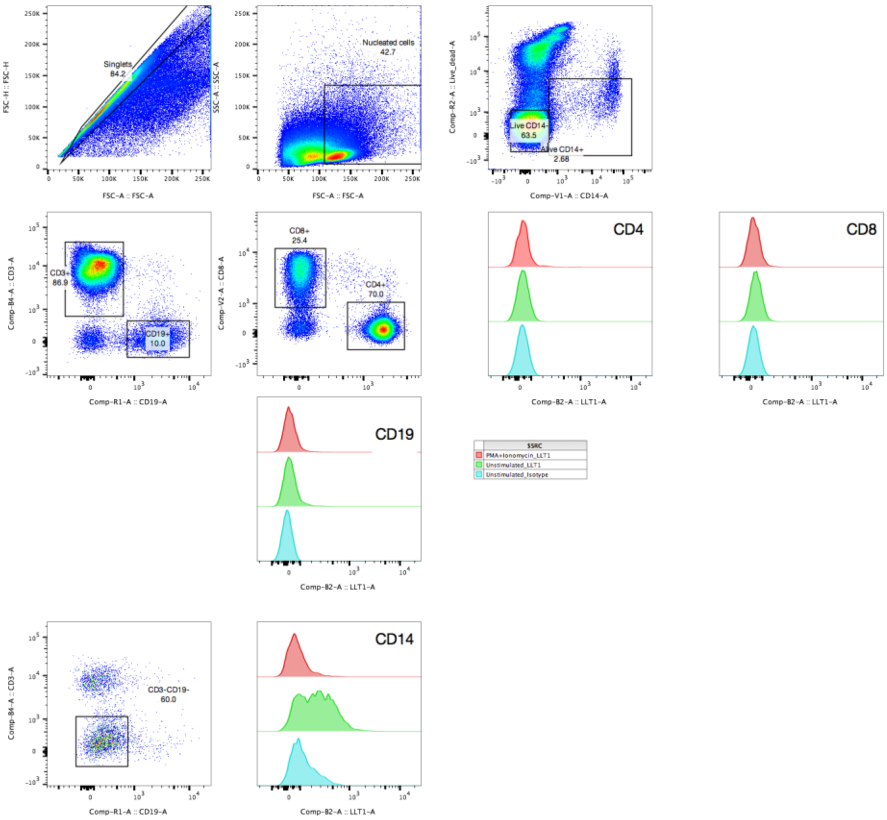
Singlets
84.2
FSC-A :: FSC-A
FSC-H :: FSC-H
Nucleated cells
42.7
SSC-A :: SSC-A
Live CD14-
63.5
Live CD14+
2.68
Comp-V1-A :: CD14-A
Comp-R2-A :: Live_dead-A
CD3+
86.9
CD19+
10.0
Comp-R1-A :: CD19-A
Comp-B4-A :: CD3-A
CD8+
25.4
CD4+
70.0
Comp-V2-A :: CD8-A
CD4
CD8
CD19
Comp-B2-A :: LLT1-A
SSRC
PMA+Ionomycin_LLT1
Unstimulated_LLT1
Unstimulated_Isotype
CD3-CD19-
60.0
CD14

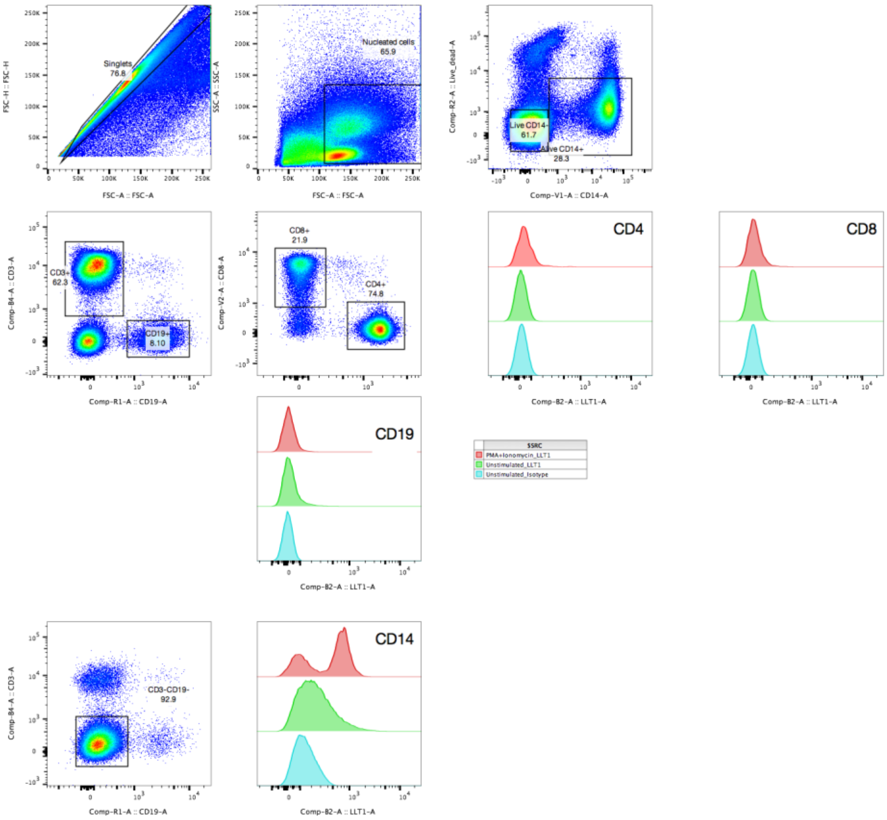
Singlets
76.8
FSC-A :: FSC-A
FSC-H :: FSC-H
Nucleated cells
65.9
SSC-A :: SSC-A
Live CD14-
61.7
Live CD14+
28.3
Comp-V1-A :: CD14-A
Comp-R2-A :: Live_dead-A
CD3+
62.3
CD19+
8.10
Comp-R1-A :: CD19-A
Comp-B4-A :: CD3-A
CD8+
21.9
CD4+
74.8
Comp-V2-A :: CD8-A
CD4
CD8
CD19
Comp-B2-A :: LLT1-A
SSRC
PMA+Ionomycin_LLT1
Unstimulated_LLT1
Unstimulated_Isotype
CD3-CD19-
92.9
CD14

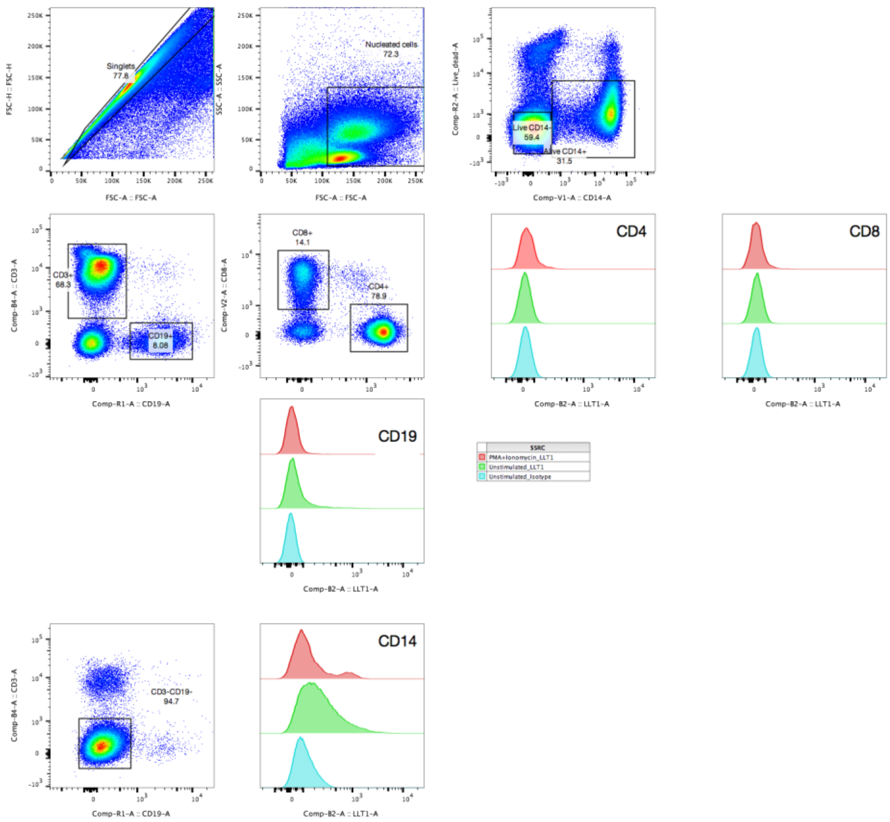
FSC-H :: FSC-H
FSC-A :: FSC-A
Singlets
77.8
SSC-A :: SSC-A
Nucleated cells
72.3
Comp-R2-A :: Live_dead-A
Comp-V1-A :: CD14-A
Live CD14-
59.4
Live CD14+
31.5
Comp-B4-A :: CD3-A
Comp-R1-A :: CD19-A
CD3+
68.3
CD19+
8.08
Comp-V2-A :: CD8-A
CD8+
14.1
CD4+
78.9
CD4
CD8
CD19
CD14
Comp-B2-A :: LLT1-A
$SRC
PMA+Ionomycin_LLT1
Unstimulated_LLT1
Unstimulated_Isotype
CD3-CD19-
94.7

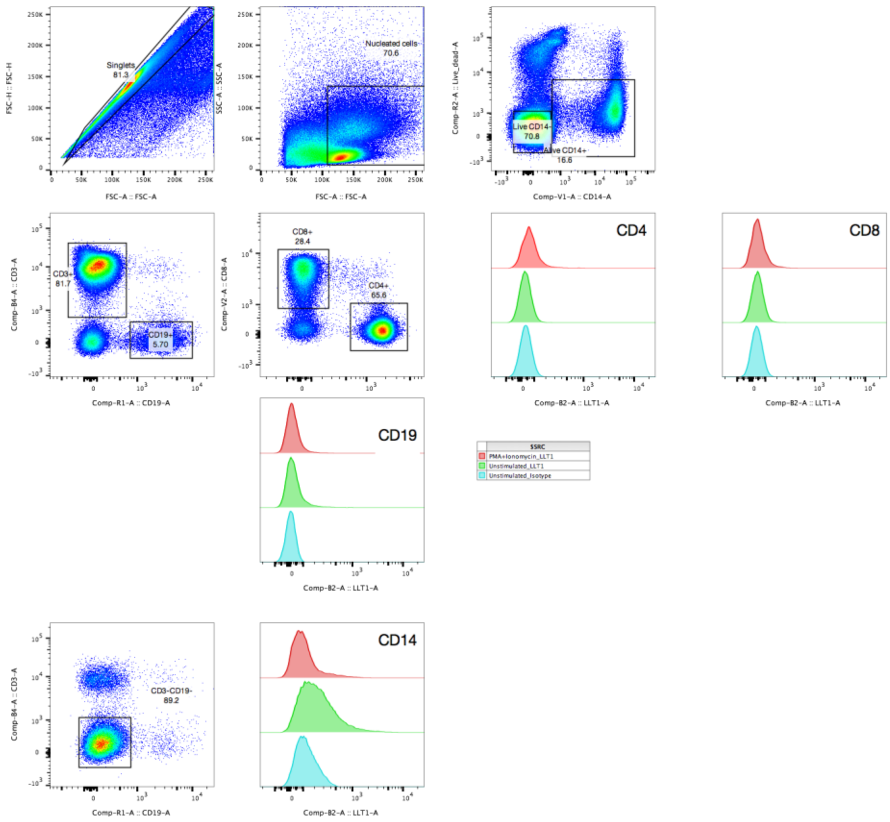
FSC-H :: FSC-H
FSC-A :: FSC-A
Singlets
81.3
SSC-A :: SSC-A
Nucleated cells
70.6
Comp-R2-A :: Live_dead-A
Comp-V1-A :: CD14-A
Live CD14-
70.8
Live CD14+
16.6
Comp-B4-A :: CD3-A
Comp-R1-A :: CD19-A
CD3+
81.7
CD19+
5.70
Comp-V2-A :: CD8-A
CD8+
28.4
CD4+
65.6
CD4
CD8
CD19
CD14
Comp-B2-A :: LLT1-A
$SRC
PMA+Ionomycin_LLT1
Unstimulated_LLT1
Unstimulated_Isotype
CD3-CD19-
89.2

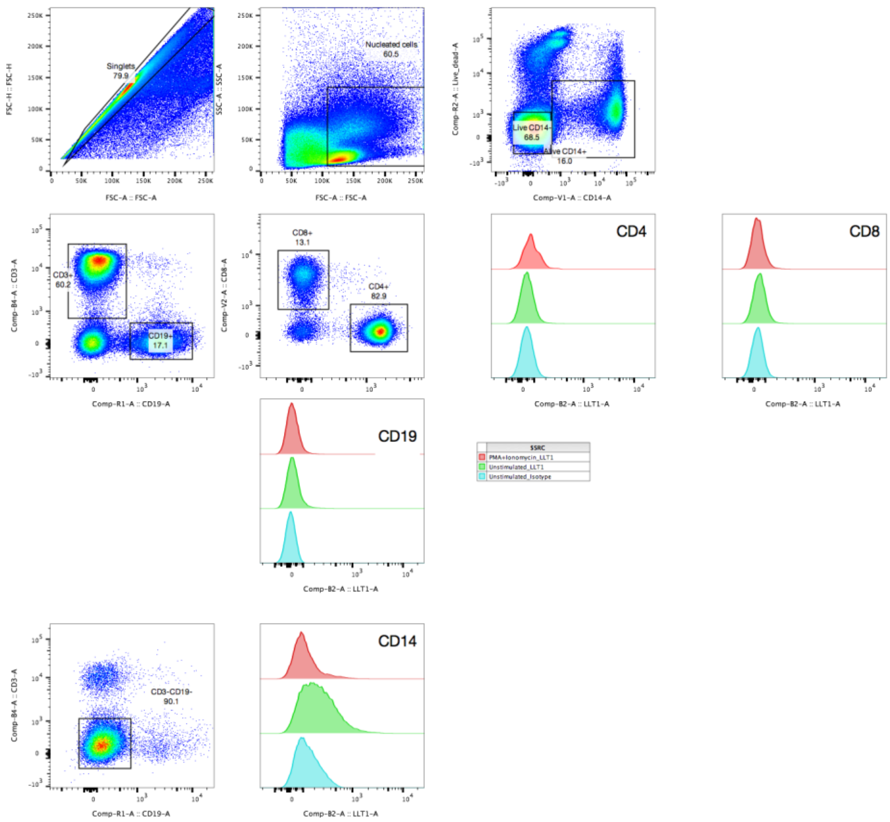
Singlets
79.9
FSC-A :: FSC-A
FSC-H :: FSC-H
Nucleated cells
60.5
SSC-A :: SSC-A
Live CD14-
66.5
Live CD14+
16.0
Comp-R2-A :: Live_dead-A
Comp-V1-A :: CD14-A
CD3+
60.2
CD19+
17.1
Comp-B4-A :: CD3-A
Comp-R1-A :: CD19-A
CD8+
13.1
CD4+
82.9
Comp-V2-A :: CD8-A
CD4
CD8
CD19
Comp-B2-A :: LLT1-A
SSRC
PMA+Ionomycin_LLT1
Unstimulated_LLT1
Unstimulated_Isotype
CD3-CD19-
90.1
CD14

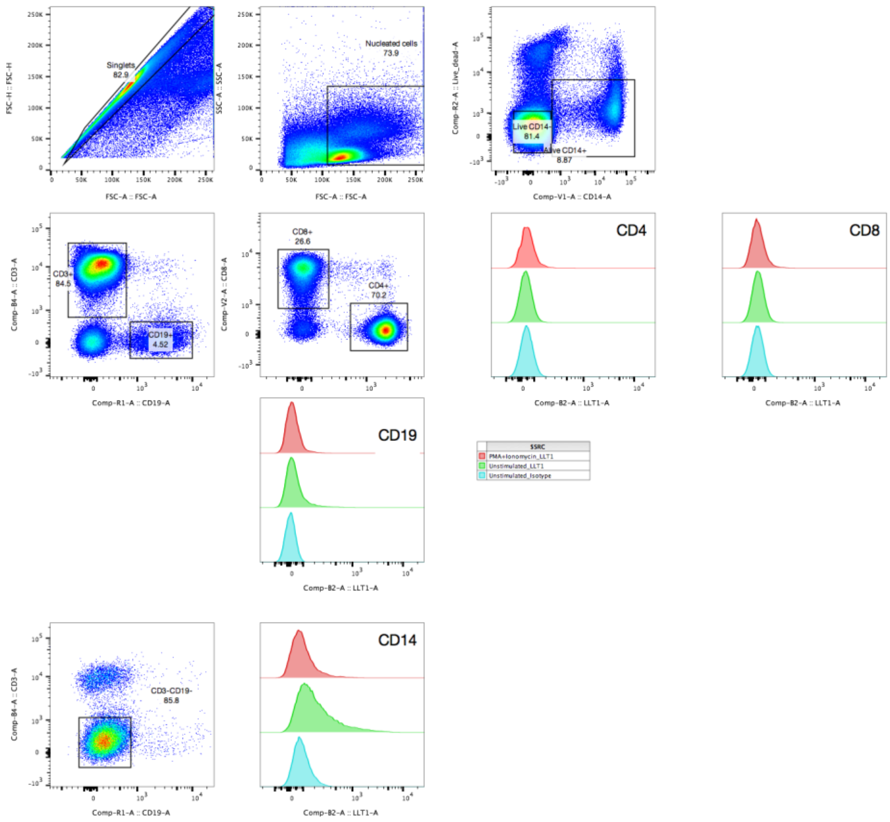
Singlets
82.9
FSC-A :: FSC-A
FSC-H :: FSC-H
Nucleated cells
73.9
SSC-A :: SSC-A
Live CD14-
81.4
Live CD14+
6.87
Comp-R2-A :: Live_dead-A
Comp-V1-A :: CD14-A
CD3+
84.5
CD19+
4.52
Comp-B4-A :: CD3-A
Comp-R1-A :: CD19-A
CD8+
26.6
CD4+
70.2
Comp-V2-A :: CD8-A
CD4
CD8
CD19
Comp-B2-A :: LLT1-A
SSRC
PMA+Ionomycin_LLT1
Unstimulated_LLT1
Unstimulated_Isotype
CD3-CD19-
85.8
CD14

PMA/Ionomycin stimulation for 48h

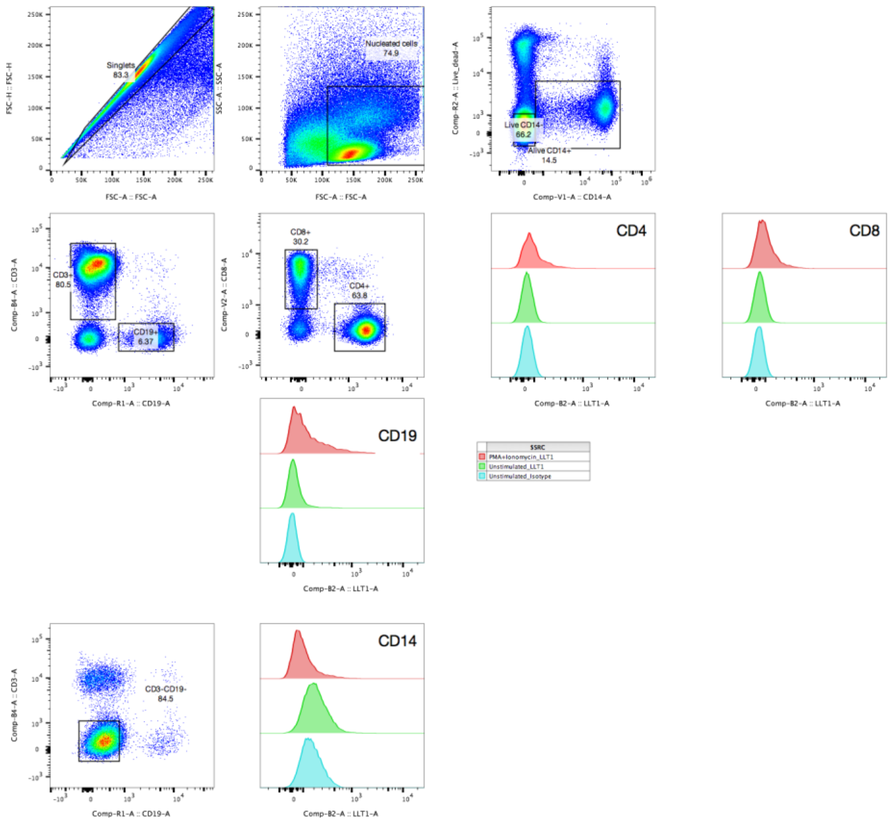

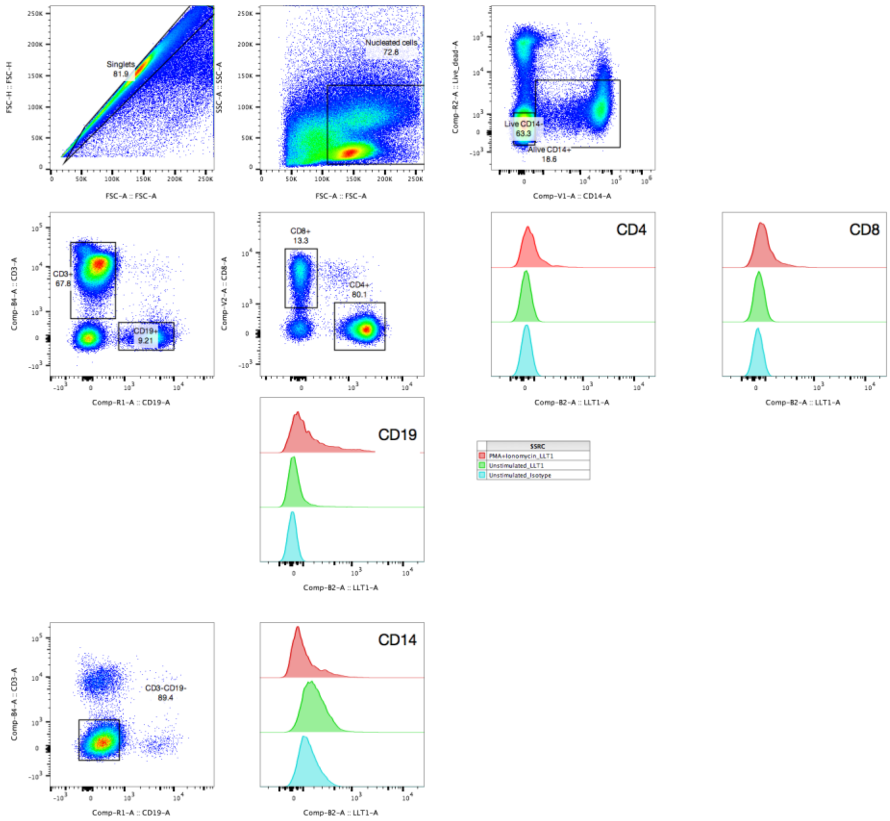

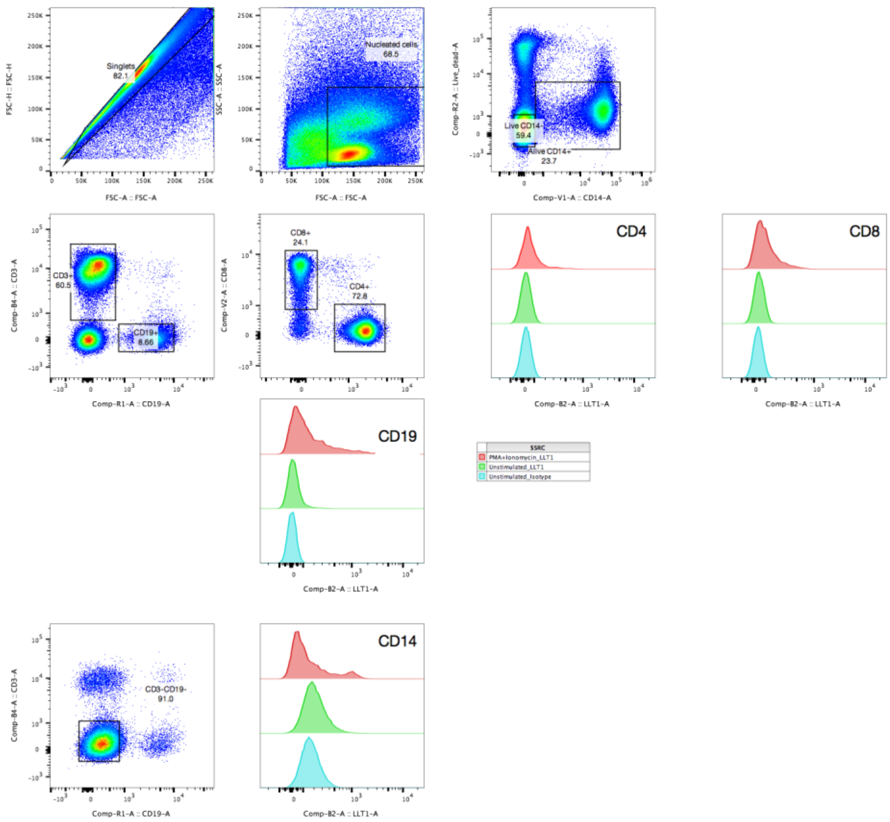
Singlets
82.1
Nucleated cells
68.5
Live CD14-
59.4
Alive CD14+
23.7
FSC-A :: FSC-A
FSC-H :: FSC-H
SSC-A :: SSC-A
Comp-V1-A :: CD14-A
Comp-R2-A :: Live_dead-A
CD3+
60.5
CD19+
8.66
Comp-R1-A :: CD19-A
Comp-B4-A :: CD3-A
CD8+
24.1
CD4+
72.8
Comp-V2-A :: CD8-A
CD4
CD8
CD19
CD14
Comp-B2-A :: LLT1-A
SSRC
PMA+Ionomycin_LLT1
Unstimulated_LLT1
Unstimulated_Isotype
CD3-CD19-
91.0

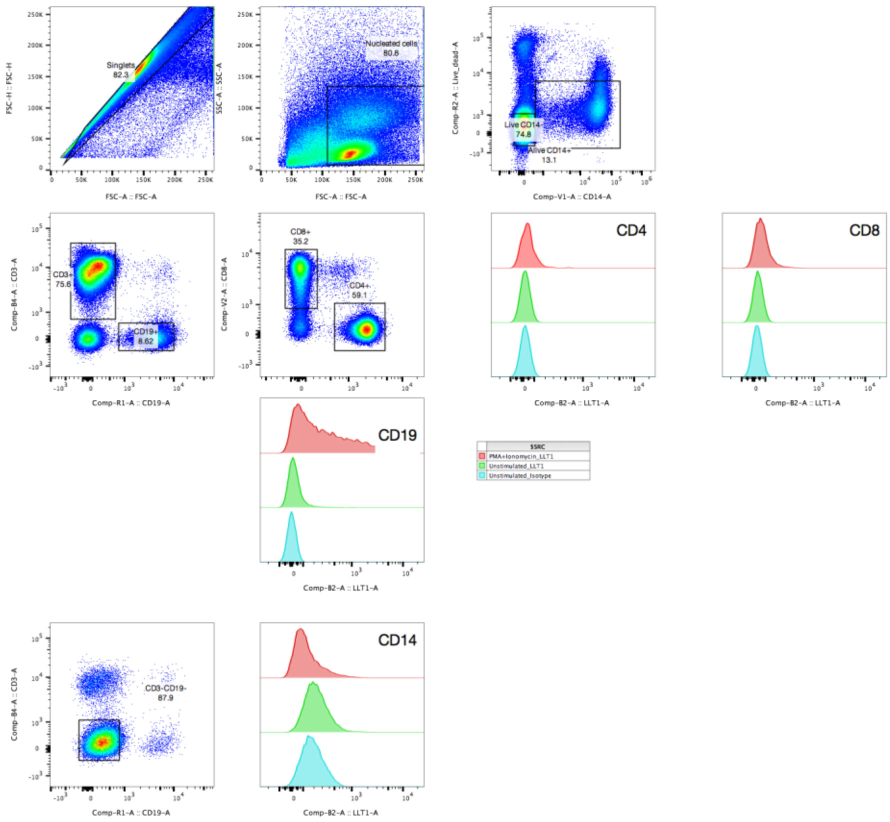
Singlets
82.3
Nucleated cells
80.8
Live CD14-
74.8
Alive CD14+
13.1
FSC-A :: FSC-A
FSC-H :: FSC-H
SSC-A :: SSC-A
Comp-V1-A :: CD14-A
Comp-R2-A :: Live_dead-A
CD3+
75.6
CD19+
8.62
Comp-R1-A :: CD19-A
Comp-B4-A :: CD3-A
CD8+
35.2
CD4+
59.1
Comp-V2-A :: CD8-A
CD4
CD8
CD19
CD14
Comp-B2-A :: LLT1-A
SSRC
PMA+Ionomycin_LLT1
Unstimulated_LLT1
Unstimulated_Isotype
CD3-CD19-
87.9

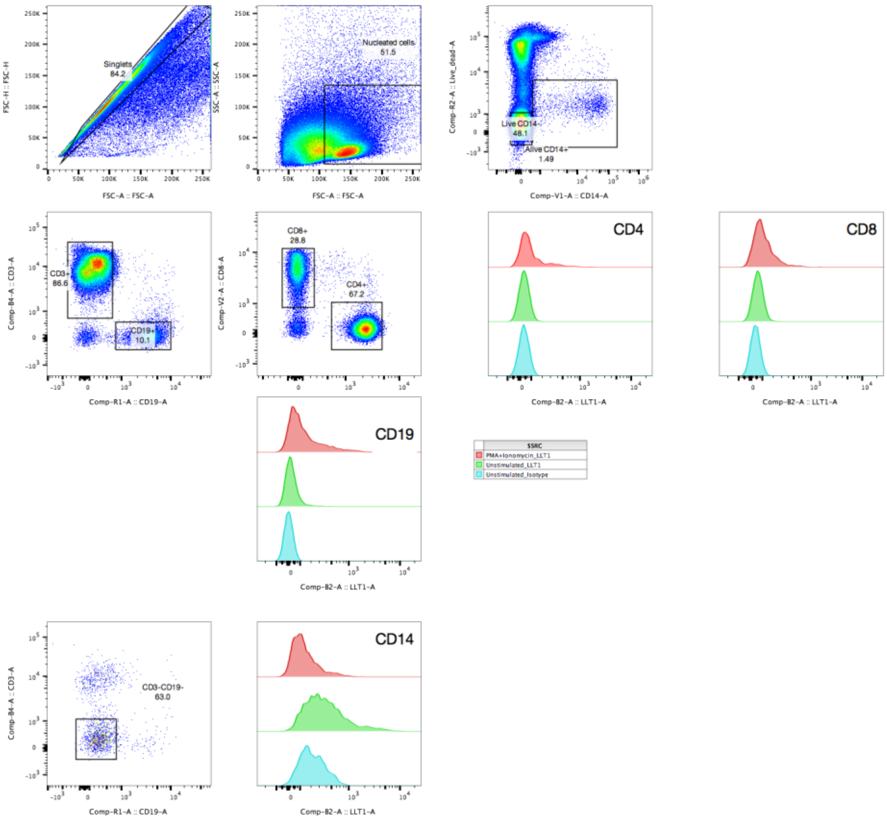
Singlets
84.2
Nucleated cells
51.5
Live CD14-
48.1
Alive CD14+
1.49
CD3+
86.6
CD19+
10.1
CD8+
28.8
CD4+
67.2
CD4
CD8
CD19
CD14
CD3-CD19-
63.0
SSRC
PMA+Ionomycin_LLT1
Unstimulated_LLT1
Unstimulated_Isotype
FSC-H :: FSC-H
FSC-A :: FSC-A
SSC-A :: SSC-A
Comp-R2-A :: Live_dead -A
Comp-V1-A :: CD14-A
Comp-B4-A :: CD3-A
Comp-R1-A :: CD19-A
Comp-V2-A :: CD8-A
Comp-B2-A :: LLT1-A

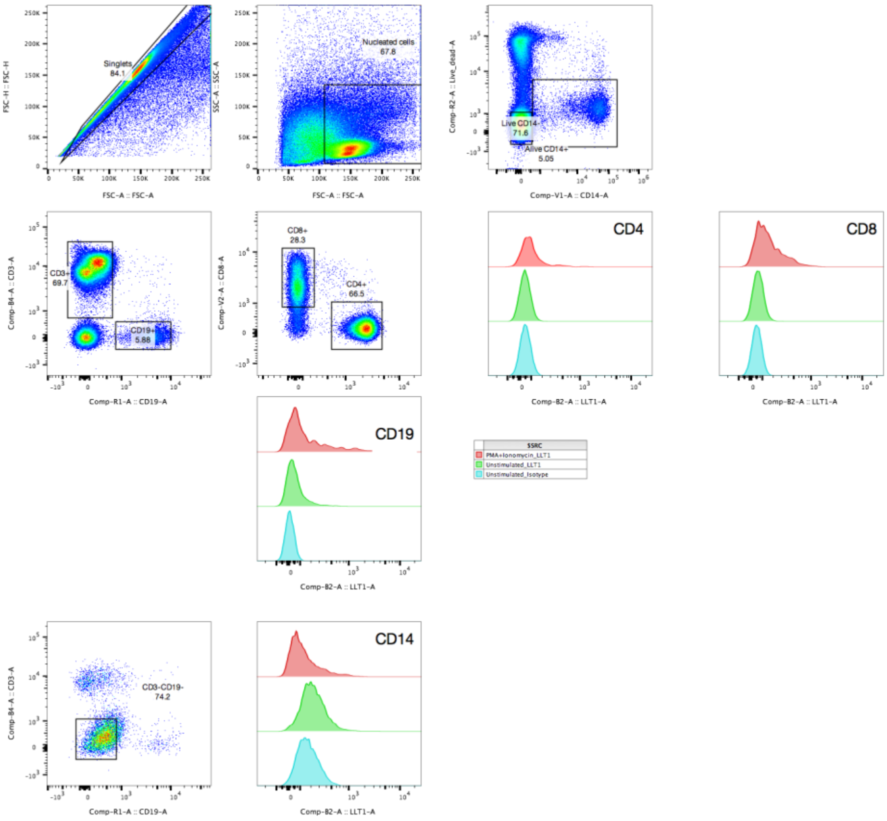
Singlets
84.1
Nucleated cells
67.8
Live CD14-
71.6
Alive CD14+
5.05
CD3+
69.7
CD19+
5.68
CD8+
28.3
CD4+
66.5
CD4
CD8
CD19
CD14
CD3-CD19-
74.2
SSRC
PMA+Ionomycin_LLT1
Unstimulated_LLT1
Unstimulated_Isotype
FSC-H :: FSC-H
FSC-A :: FSC-A
SSC-A :: SSC-A
Comp-R2-A :: Live_dead -A
Comp-V1-A :: CD14-A
Comp-B4-A :: CD3-A
Comp-R1-A :: CD19-A
Comp-V2-A :: CD8-A
Comp-B2-A :: LLT1-A

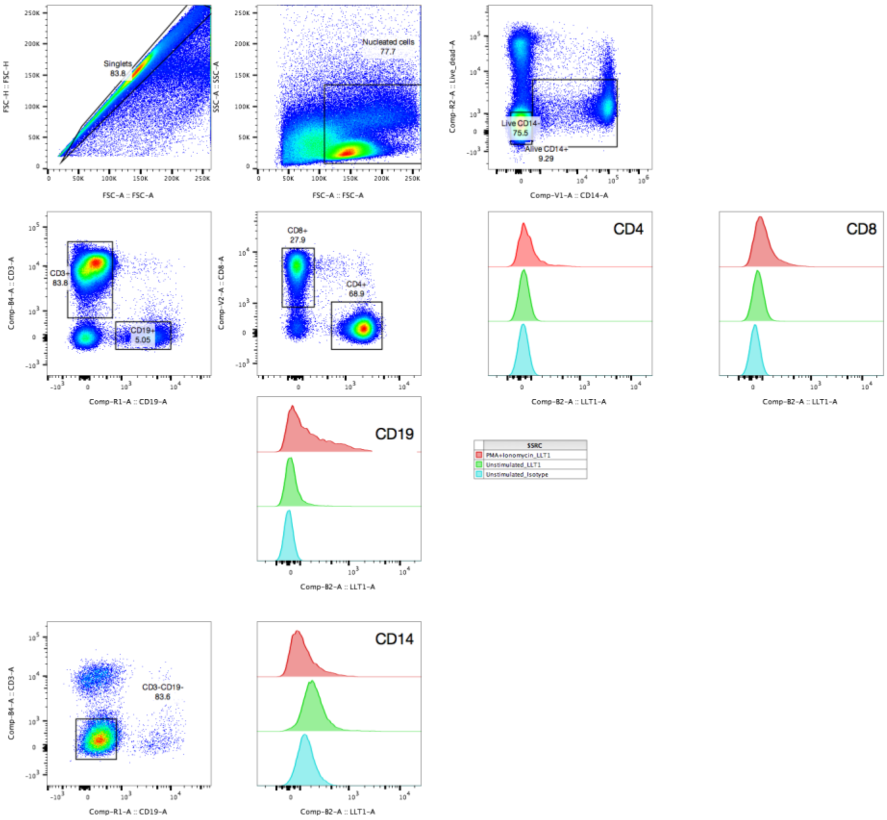
Singlets
83.8
FSC-H :: FSC-H
FSC-A :: FSC-A
Nucleated cells
77.7
SSC-A :: SSC-A
Comp-R2-A :: Live_dead-A
Live CD14-
75.5
Alive CD14+
9.29
Comp-V1-A :: CD14-A
Comp-B4-A :: CD3-A
CD3+
83.8
CD19+
5.05
Comp-R1-A :: CD19-A
Comp-V2-A :: CD8-A
CD8+
27.9
CD4+
68.9
CD4
CD8
Comp-B2-A :: LLT1-A
CD19
SSRC
PMA+Ionomycin_LLT1
Unstimulated_LLT1
Unstimulated_Isotype
CD3-CD19-
83.6
CD14

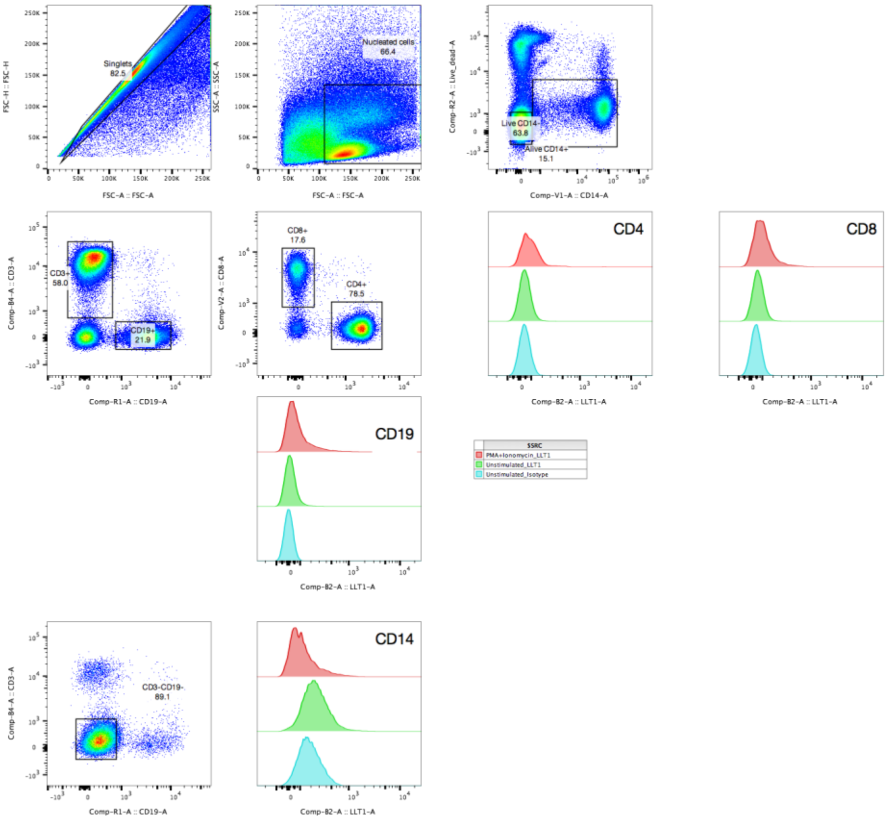
Singlets
82.5
FSC-H :: FSC-H
FSC-A :: FSC-A
Nucleated cells
66.4
SSC-A :: SSC-A
Comp-R2-A :: Live_dead-A
Live CD14-
63.8
Alive CD14+
15.1
Comp-V1-A :: CD14-A
Comp-B4-A :: CD3-A
CD3+
58.0
CD19+
21.9
Comp-R1-A :: CD19-A
Comp-V2-A :: CD8-A
CD8+
17.6
CD4+
78.5
CD4
CD8
Comp-B2-A :: LLT1-A
CD19
SSRC
PMA+Ionomycin_LLT1
Unstimulated_LLT1
Unstimulated_Isotype
CD3-CD19-
89.1
CD14

PHA stimulation after 24h

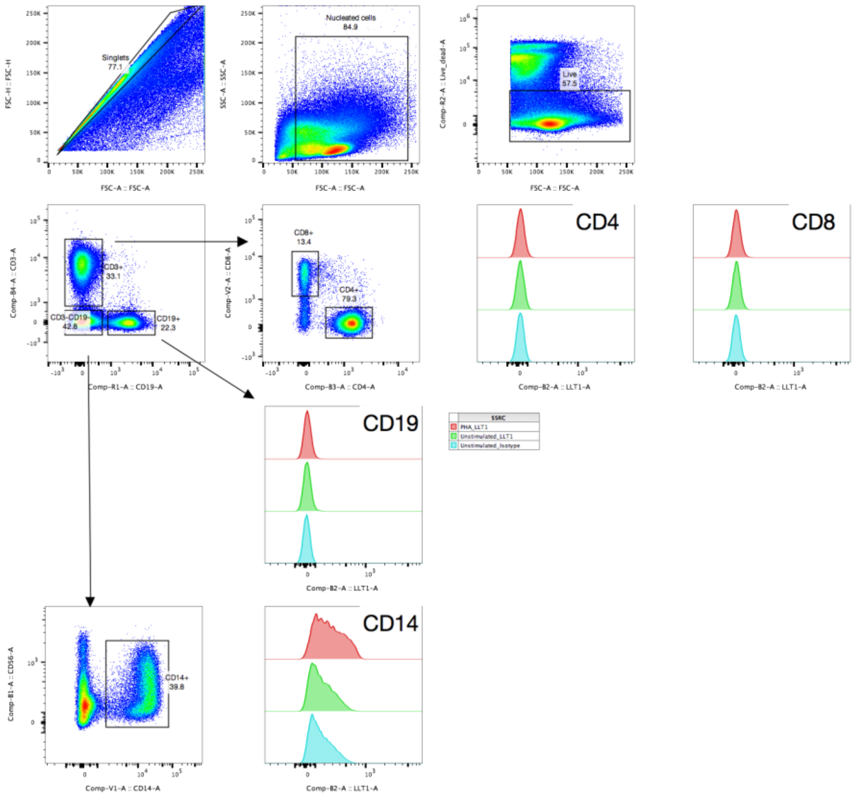

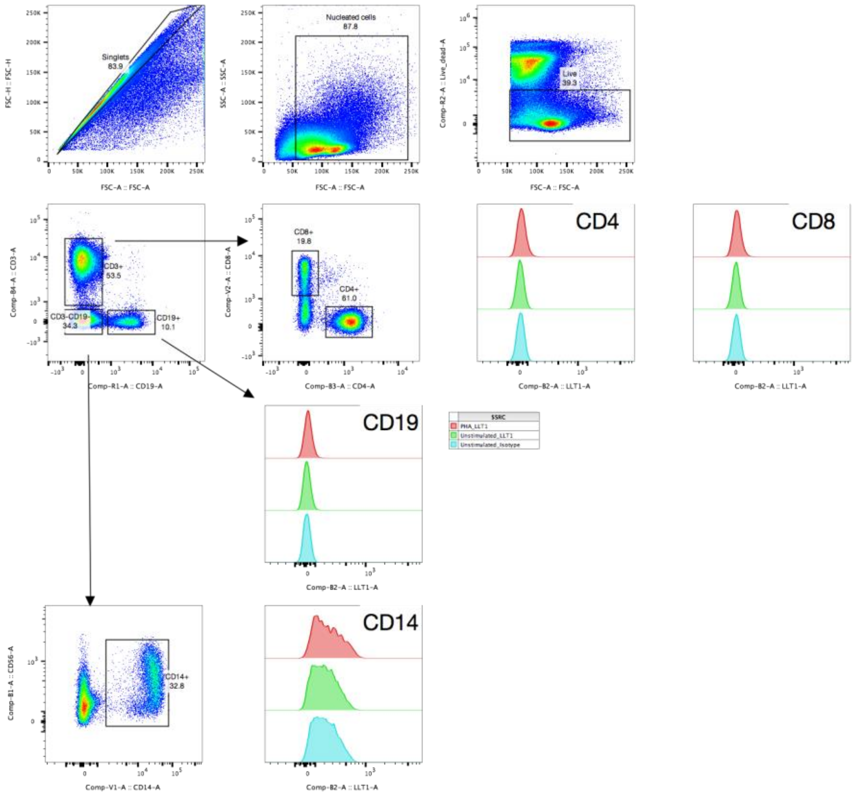

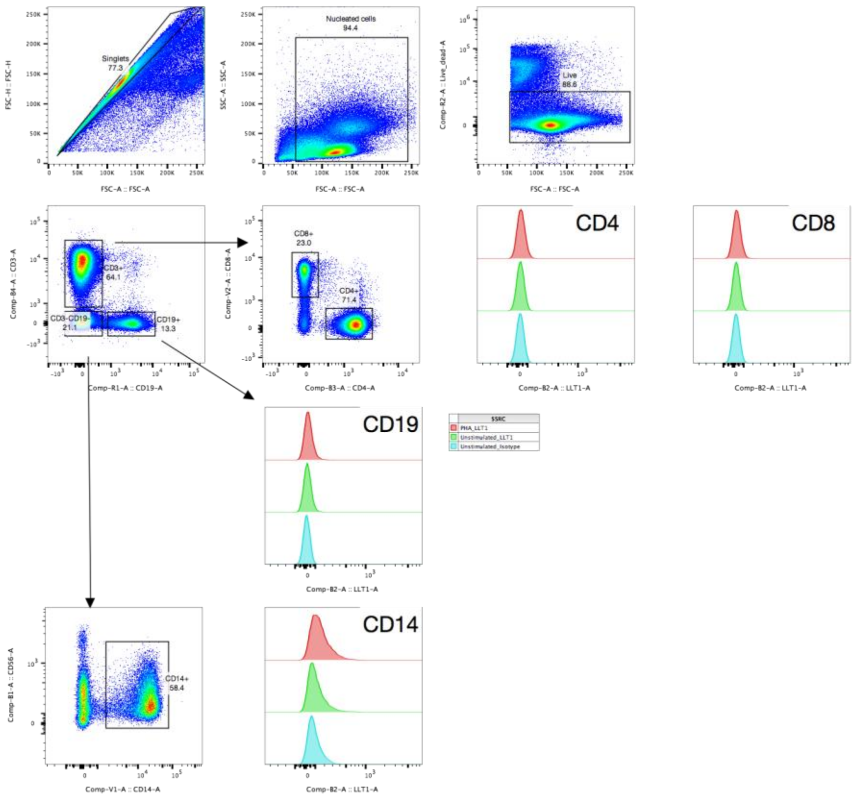
Singlets
77.3
FSC-H :: FSC-H
FSC-A :: FSC-A
Nucleated cells
94.4
SSC-A :: SSC-A
Live
88.6
Comp-R2-A :: Live_dead-A
CD3+
64.1
CD3-CD19-
21.1
CD19+
13.3
Comp-B4-A :: CD3-A
Comp-R1-A :: CD19-A
CD8+
23.0
CD4+
71.4
Comp-V2-A :: CD8-A
Comp-B3-A :: CD4-A
CD4
CD8
Comp-B2-A :: LLT1-A
CD19
SSRC
PHA_LLT1
Unstimulated_LLT1
Unstimulated_Isotype
CD14+
58.4
Comp-B1-A :: CD56-A
Comp-V1-A :: CD14-A
CD14

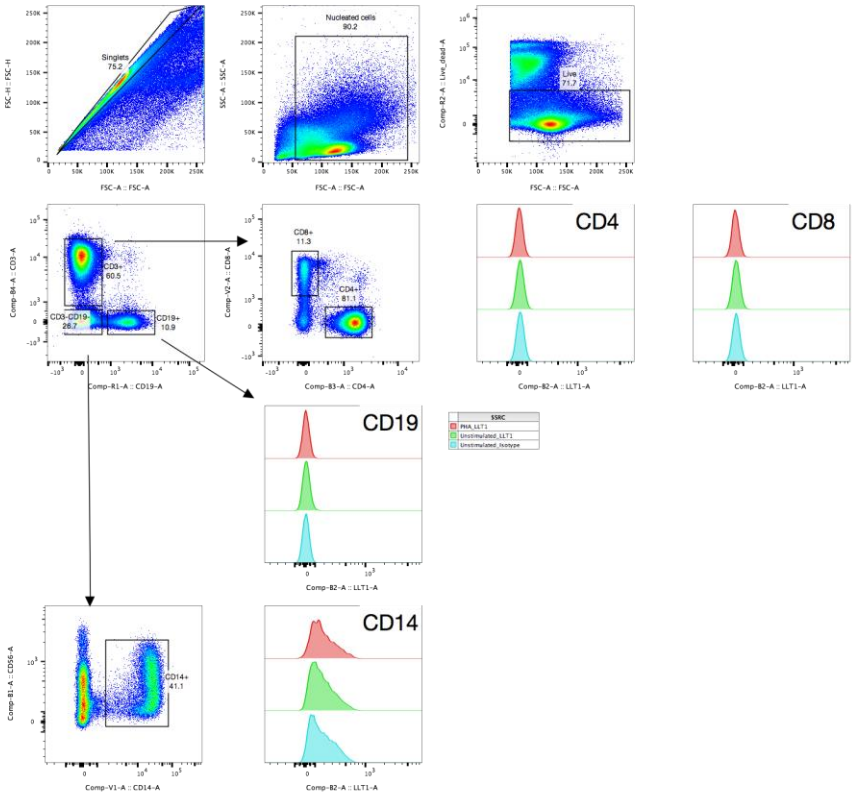
Singlets
75.2
FSC-H :: FSC-H
FSC-A :: FSC-A
Nucleated cells
90.2
SSC-A :: SSC-A
Live
71.7
Comp-R2-A :: Live_dead-A
CD3+
60.5
CD3-CD19-
26.7
CD19+
10.9
Comp-B4-A :: CD3-A
Comp-R1-A :: CD19-A
CD8+
11.3
CD4+
81.1
Comp-V2-A :: CD8-A
Comp-B3-A :: CD4-A
CD4
CD8
Comp-B2-A :: LLT1-A
CD19
SSRC
PHA_LLT1
Unstimulated_LLT1
Unstimulated_Isotype
CD14+
41.1
Comp-B1-A :: CD56-A
Comp-V1-A :: CD14-A
CD14

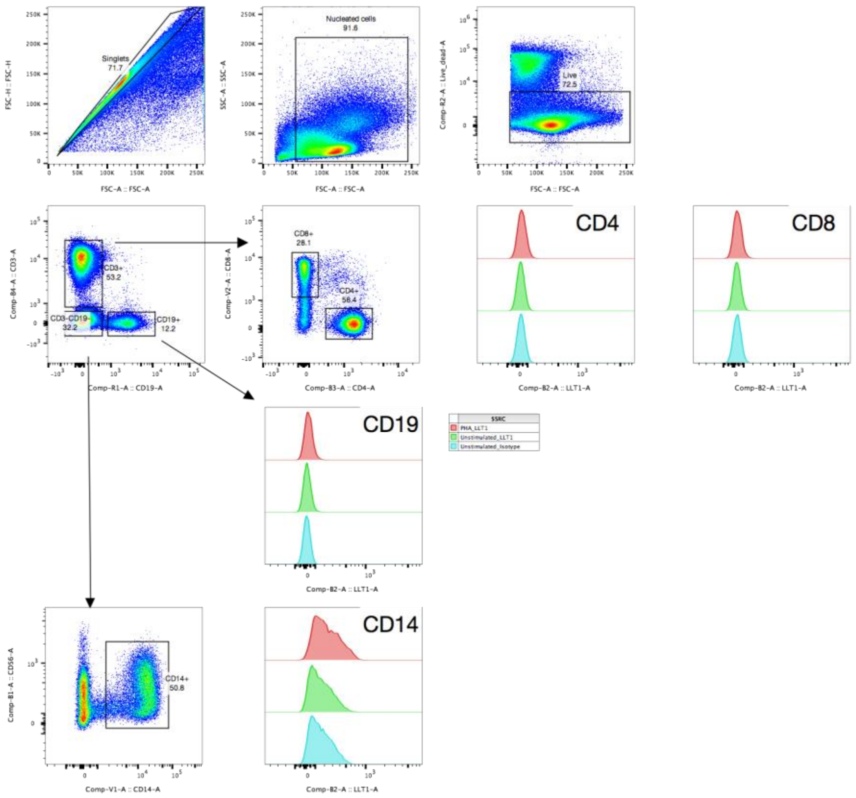
Singlets
71.7
FSC-H :: FSC-H
FSC-A :: FSC-A
Nucleated cells
91.8
SSC-A :: SSC-A
Live
72.5
Comp-R2-A :: Live_dead-A
Comp-B4-A :: CD3-A
CD3+
53.2
CD3-CD19-
32.2
CD19+
12.2
Comp-R1-A :: CD19-A
Comp-V2-A :: CD8-A
CD8+
28.1
CD4+
56.4
Comp-B3-A :: CD4-A
CD4
CD8
Comp-B2-A :: LLT1-A
CD19
SSRC
PHA_LLT1
Unstimulated_LLT1
Unstimulated_Isotype
Comp-B1-A :: CD56-A
CD14+
50.8
Comp-V1-A :: CD14-A
CD14

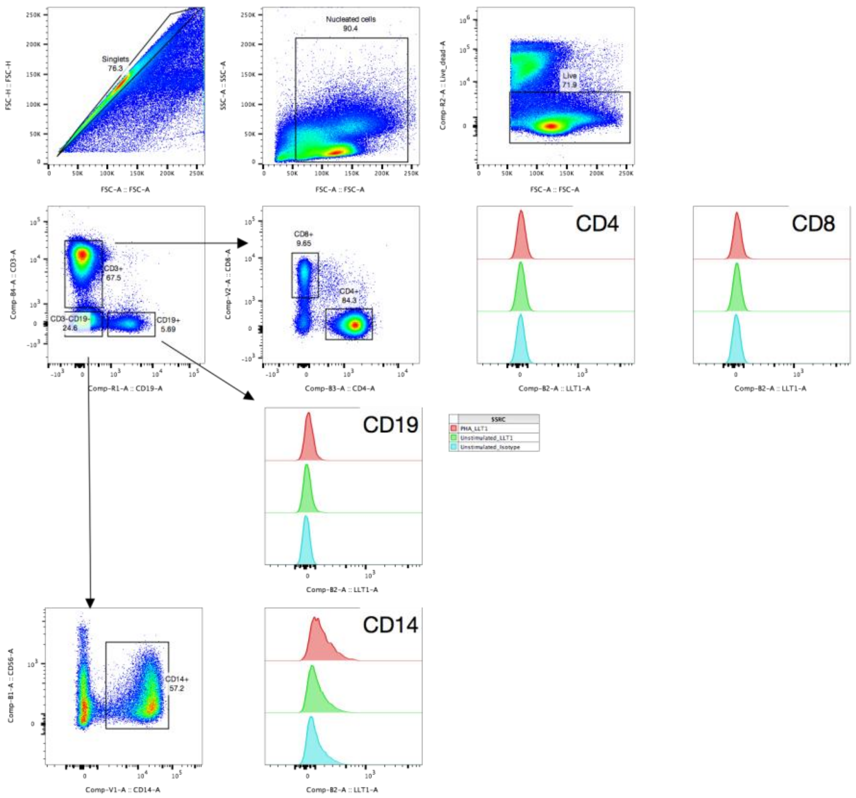
Singlets
76.3
FSC-H :: FSC-H
FSC-A :: FSC-A
Nucleated cells
90.4
SSC-A :: SSC-A
Live
71.9
Comp-R2-A :: Live_dead-A
Comp-B4-A :: CD3-A
CD3+
67.5
CD3-CD19-
24.6
CD19+
5.89
Comp-R1-A :: CD19-A
Comp-V2-A :: CD8-A
CD8+
9.65
CD4+
84.3
Comp-B3-A :: CD4-A
CD4
CD8
Comp-B2-A :: LLT1-A
CD19
SSRC
PHA_LLT1
Unstimulated_LLT1
Unstimulated_Isotype
Comp-B1-A :: CD56-A
CD14+
57.2
Comp-V1-A :: CD14-A
CD14

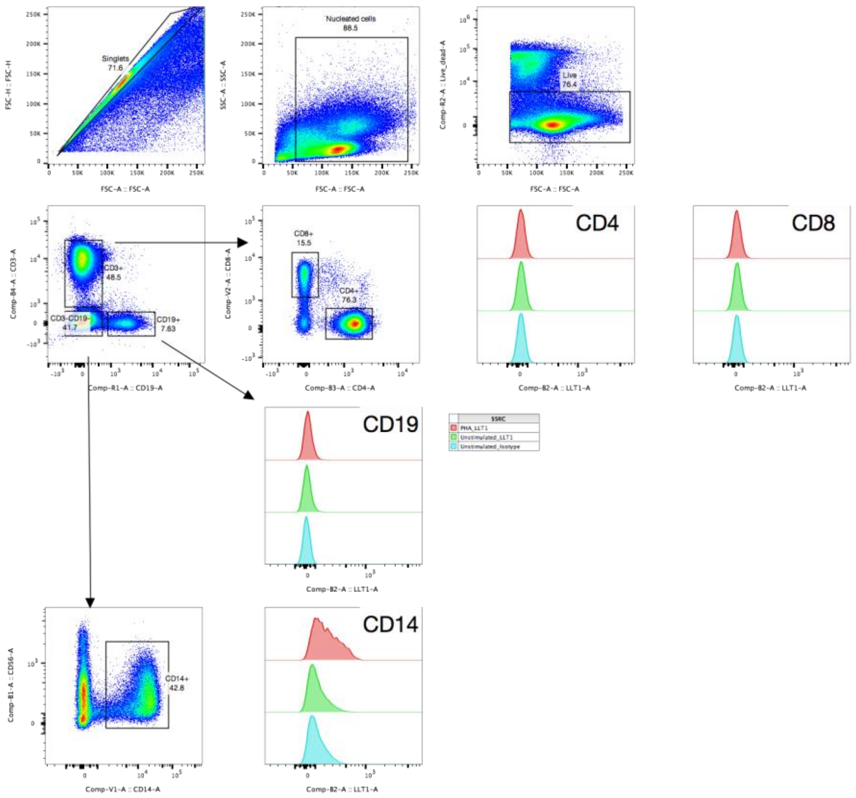
Singlets
71.6
FSC-H :: FSC-H
FSC-A :: FSC-A
Nucleated cells
88.5
SSC-A :: SSC-A
Live
76.4
Comp-R2-A :: Live_dead-A
CD3+
48.5
CD3-CD19-
41.7
CD19+
7.63
Comp-B4-A :: CD3-A
Comp-R1-A :: CD19-A
CD8+
15.5
CD4+
76.3
Comp-V2-A :: CD8-A
Comp-B3-A :: CD4-A
CD4
CD8
Comp-B2-A :: LLT1-A
CD19
SSRC
PHA_LLT1
Unstimulated_LLT1
Unstimulated_Isotype
CD14+
42.8
Comp-B1-A :: CD56-A
Comp-V1-A :: CD14-A
CD14

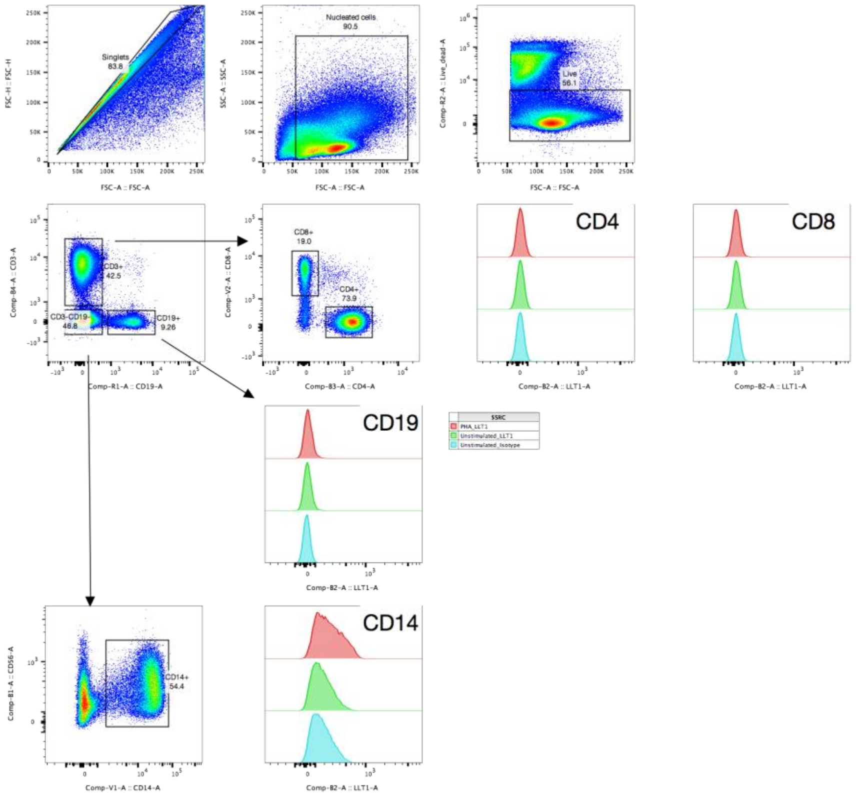
Singlets
83.8
FSC-H :: FSC-H
FSC-A :: FSC-A
Nucleated cells
90.5
SSC-A :: SSC-A
Live
56.1
Comp-R2-A :: Live_dead-A
CD3+
42.5
CD3-CD19-
46.8
CD19+
9.26
Comp-B4-A :: CD3-A
Comp-R1-A :: CD19-A
CD8+
19.0
CD4+
73.9
Comp-V2-A :: CD8-A
Comp-B3-A :: CD4-A
CD4
CD8
Comp-B2-A :: LLT1-A
CD19
SSRC
PHA_LLT1
Unstimulated_LLT1
Unstimulated_Isotype
CD14+
54.4
Comp-B1-A :: CD56-A
Comp-V1-A :: CD14-A
CD14

PHA stimulation after 48h

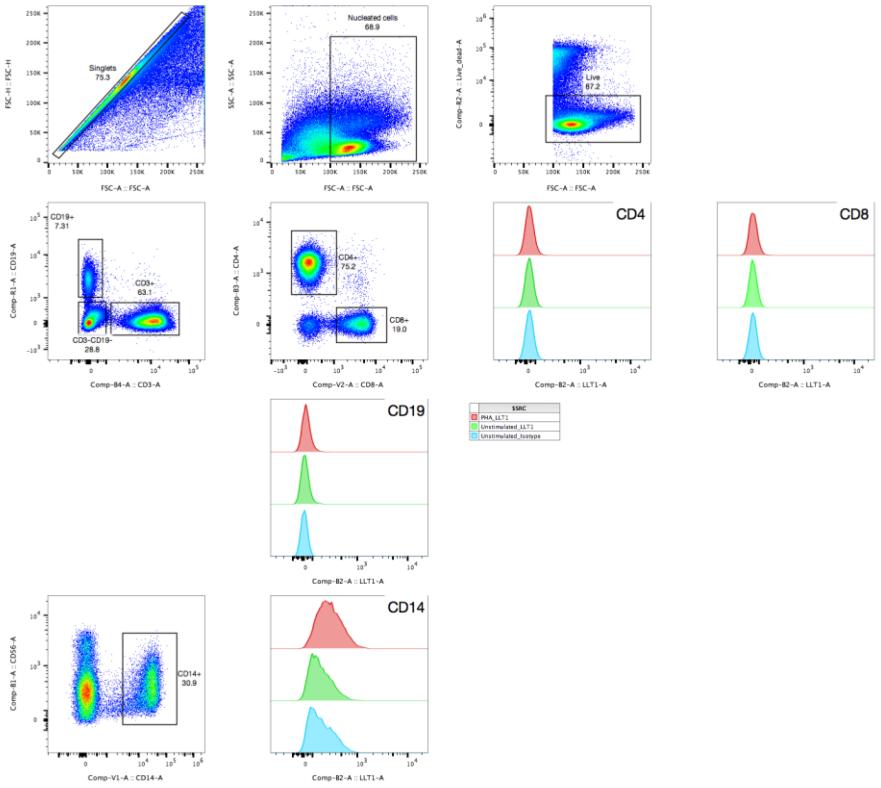

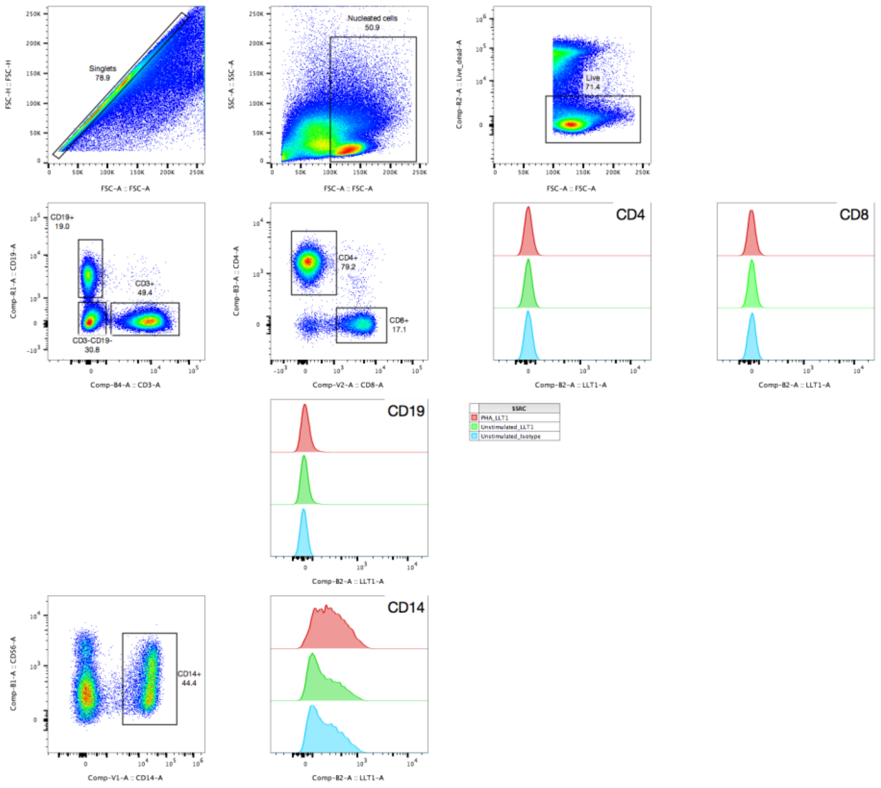

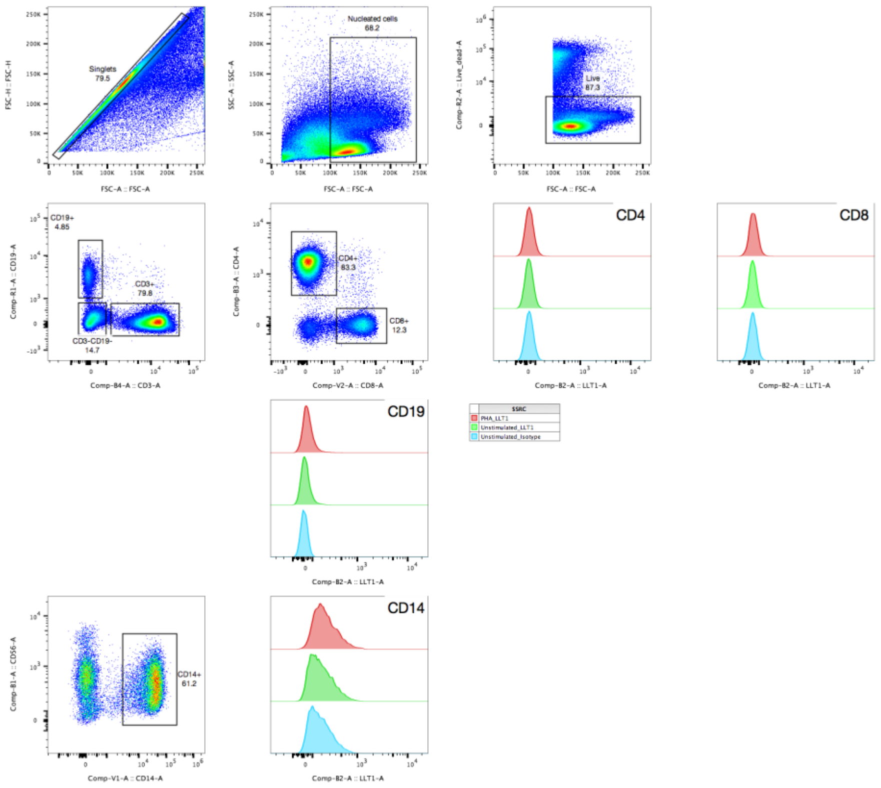
Singlets
79.5
Nucleated cells
68.2
Live
87.3
FSC-A :: FSC-A
FSC-H :: FSC-H
SSC-A :: SSC-A
Comp-R2-A :: Live_dead-A
CD19+
4.85
CD3+
79.8
CD3-CD19-
14.7
Comp-R1-A :: CD19-A
Comp-B4-A :: CD3-A
CD4+
83.3
CD8+
12.3
Comp-B3-A :: CD4-A
Comp-V2-A :: CD8-A
CD4
CD8
CD19
CD14
Comp-B2-A :: LLT1-A
SSRC
PHA_LLT1
Unstimulated_LLT1
Unstimulated_Isotype
CD14+
61.2
Comp-B1-A :: CD56-A
Comp-V1-A :: CD14-A

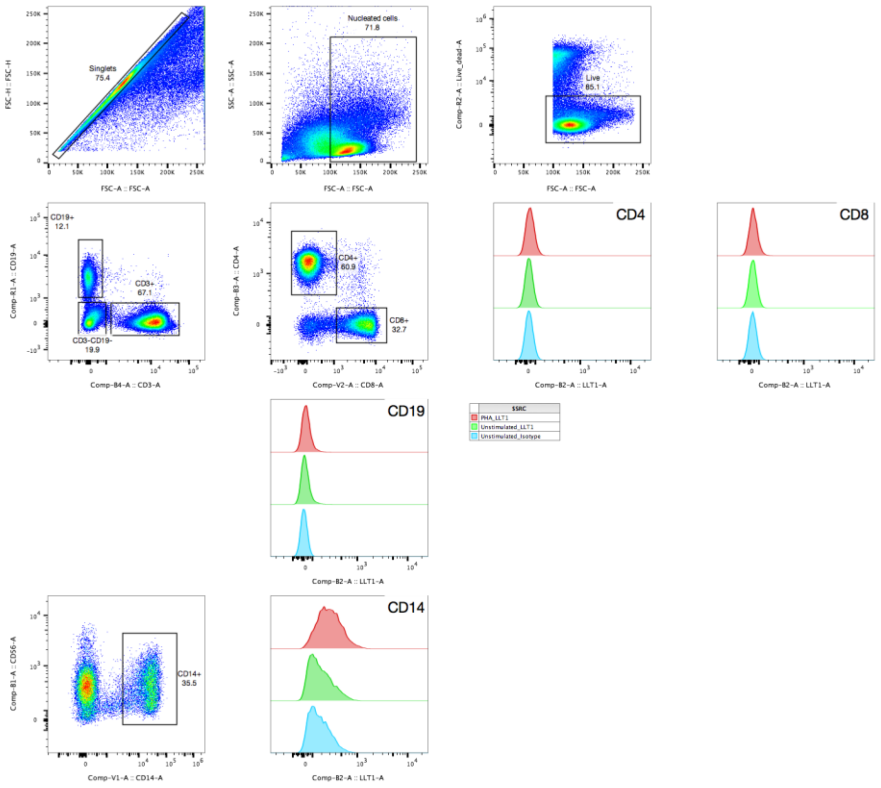
Singlets
75.4
Nucleated cells
71.8
Live
85.1
FSC-A :: FSC-A
FSC-H :: FSC-H
SSC-A :: SSC-A
Comp-R2-A :: Live_dead-A
CD19+
12.1
CD3+
67.1
CD3-CD19-
19.9
Comp-R1-A :: CD19-A
Comp-B4-A :: CD3-A
CD4+
80.9
CD8+
32.7
Comp-B3-A :: CD4-A
Comp-V2-A :: CD8-A
CD4
CD8
CD19
CD14
Comp-B2-A :: LLT1-A
SSRC
PHA_LLT1
Unstimulated_LLT1
Unstimulated_Isotype
CD14+
35.5
Comp-B1-A :: CD56-A
Comp-V1-A :: CD14-A

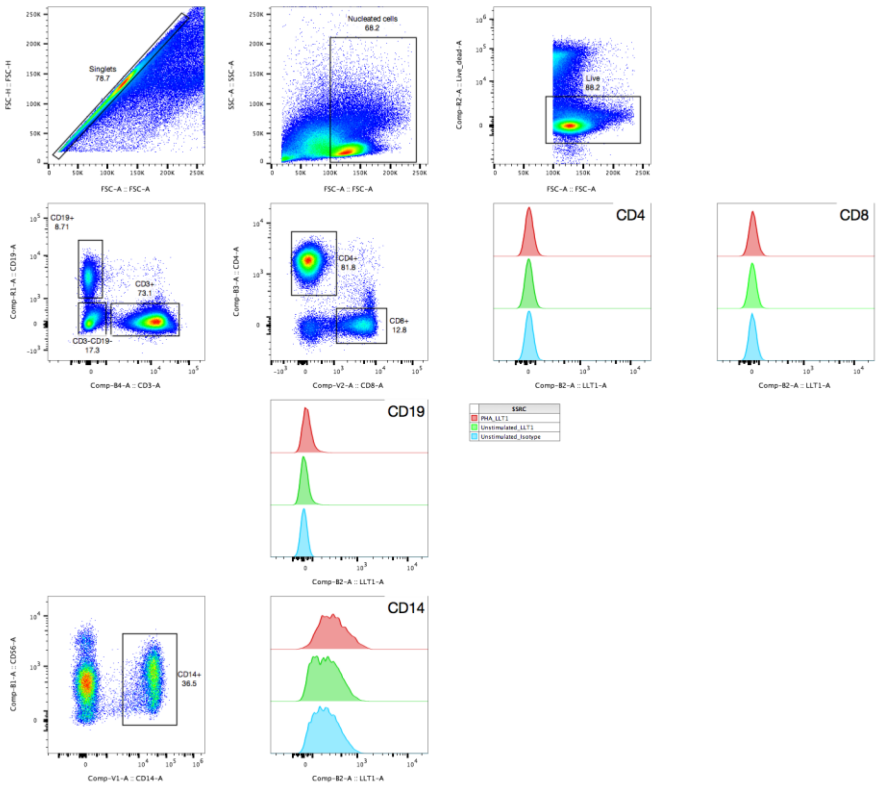
Singlets
78.7
FSC-A :: FSC-A
FSC-H :: FSC-H
Nucleated cells
68.2
SSC-A :: SSC-A
Live
88.2
Comp-R2-A :: Live_dead-A
CD19+
8.71
CD3+
73.1
CD3-CD19-
17.3
Comp-R1-A :: CD19-A
Comp-B4-A :: CD3-A
CD4+
81.8
CD8+
12.8
Comp-B3-A :: CD4-A
Comp-V2-A :: CD8-A
CD4
CD8
CD19
CD14
Comp-B2-A :: LLT1-A
$SRC
PHA_LLT1
Unstimulated_LLT1
Unstimulated_Isotype
CD14+
36.5
Comp-B1-A :: CD56-A
Comp-V1-A :: CD14-A

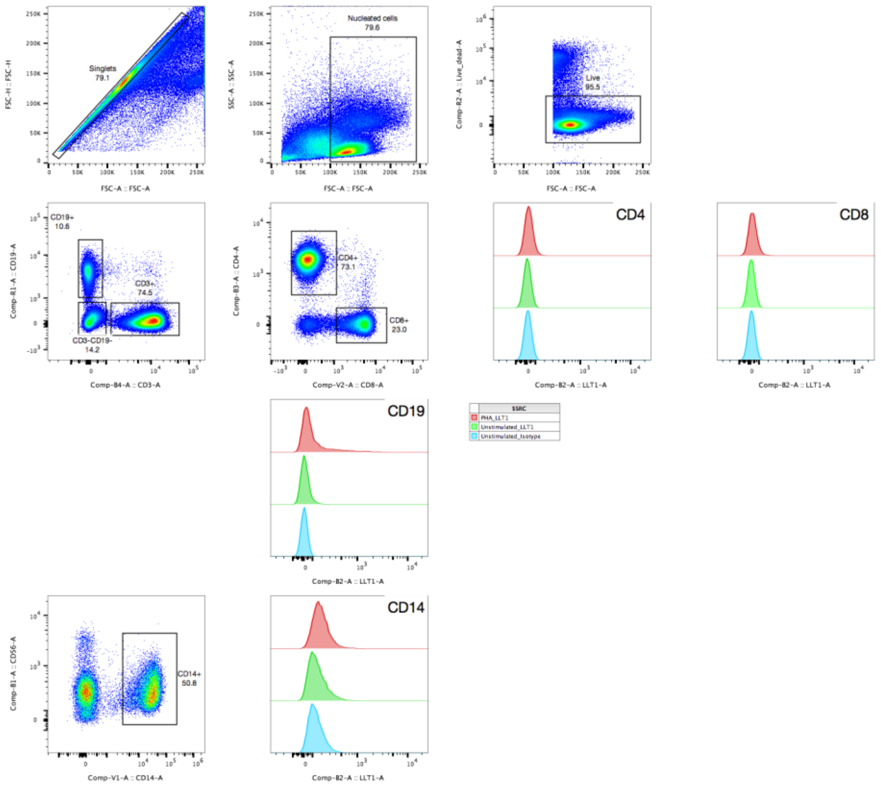
Singlets
79.1
FSC-A :: FSC-A
FSC-H :: FSC-H
Nucleated cells
79.6
SSC-A :: SSC-A
Live
95.5
Comp-R2-A :: Live_dead-A
CD19+
10.6
CD3+
74.5
CD3-CD19-
14.2
Comp-R1-A :: CD19-A
Comp-B4-A :: CD3-A
CD4+
73.1
CD8+
23.0
Comp-B3-A :: CD4-A
Comp-V2-A :: CD8-A
CD4
CD8
CD19
CD14
Comp-B2-A :: LLT1-A
$SRC
PHA_LLT1
Unstimulated_LLT1
Unstimulated_Isotype
CD14+
50.8
Comp-B1-A :: CD56-A
Comp-V1-A :: CD14-A

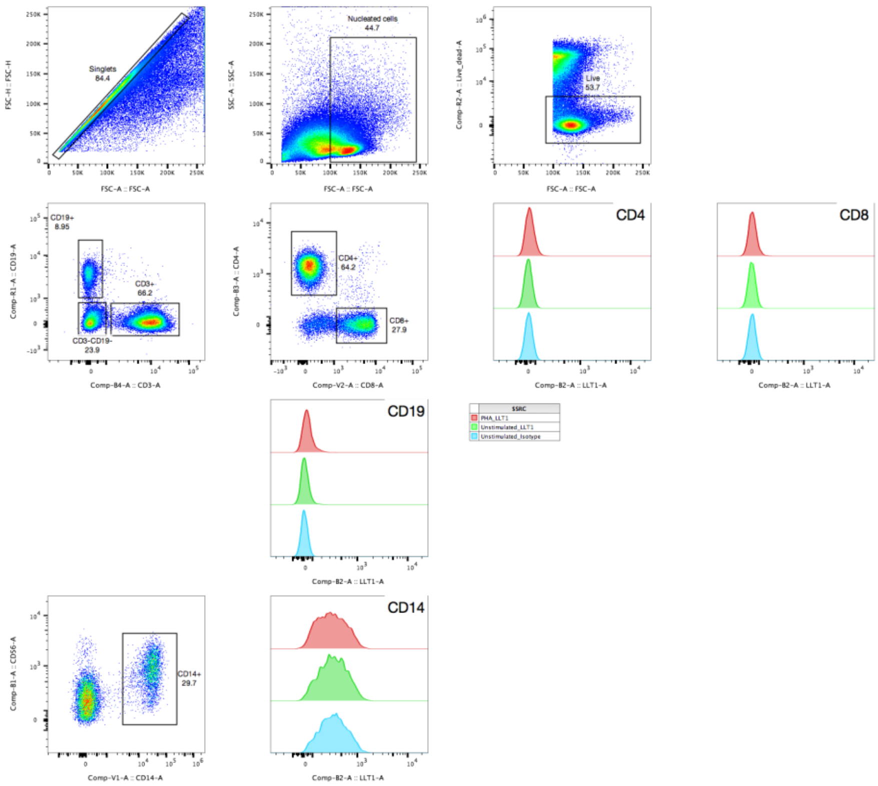
Singlets
84.4
Nucleated cells
44.7
Live
53.7
CD19+
8.95
CD3+
66.2
CD3-CD19-
23.9
CD4+
64.2
CD8+
27.9
CD4
CD8
CD19
CD14
CD14+
29.7
$SRC
PHA_LLT1
Unstimulated_LLT1
Unstimulated_Isotype
FSC-A :: FSC-A
FSC-H :: FSC-H
SSC-A :: SSC-A
Comp-B2-A :: Live_dead-A
Comp-R1-A :: CD19-A
Comp-B4-A :: CD3-A
Comp-B3-A :: CD4-A
Comp-V2-A :: CD8-A
Comp-B2-A :: LLT1-A
Comp-B1-A :: CD56-A
Comp-V1-A :: CD14-A

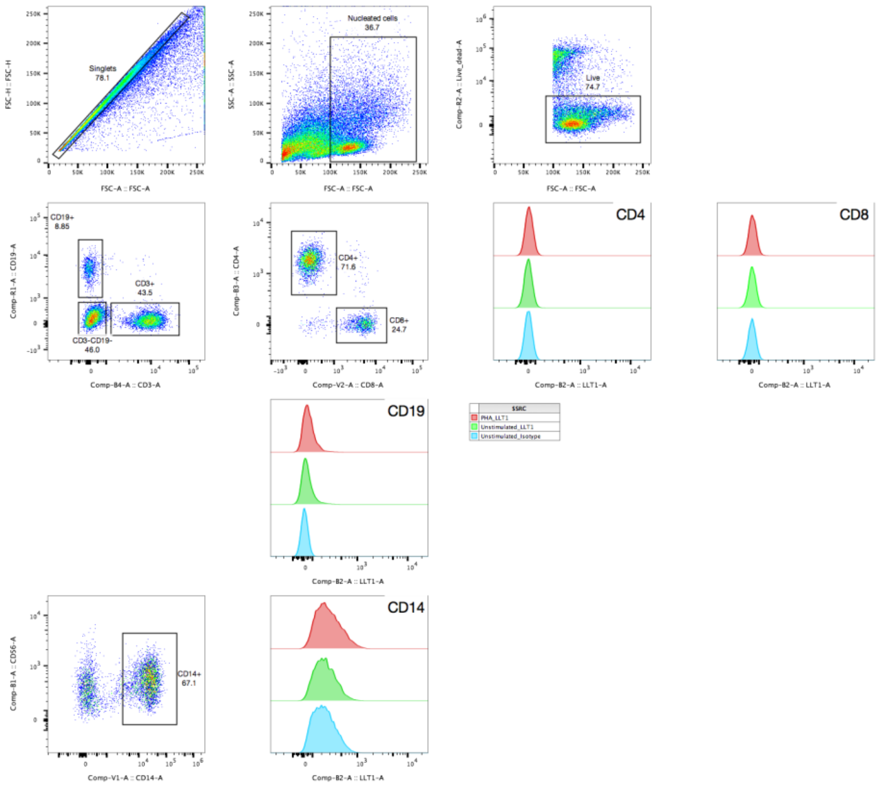
Singlets
78.1
Nucleated cells
36.7
Live
74.7
CD19+
8.85
CD3+
43.5
CD3-CD19-
46.0
CD4+
71.6
CD8+
24.7
CD4
CD8
CD19
CD14
CD14+
67.1
$SRC
PHA_LLT1
Unstimulated_LLT1
Unstimulated_Isotype
FSC-A :: FSC-A
FSC-H :: FSC-H
SSC-A :: SSC-A
Comp-B2-A :: Live_dead-A
Comp-R1-A :: CD19-A
Comp-B4-A :: CD3-A
Comp-B3-A :: CD4-A
Comp-V2-A :: CD8-A
Comp-B2-A :: LLT1-A
Comp-B1-A :: CD56-A
Comp-V1-A :: CD14-A

Expression of lectin-like transcript 1 (LLT1) in healthy immune tissue. Staining of human healthy tissue with the 2H7 anti-LLT1 antibody at 1/500. 5x, 10x and 20x magnification (all stainings undertaken for Figure 3).

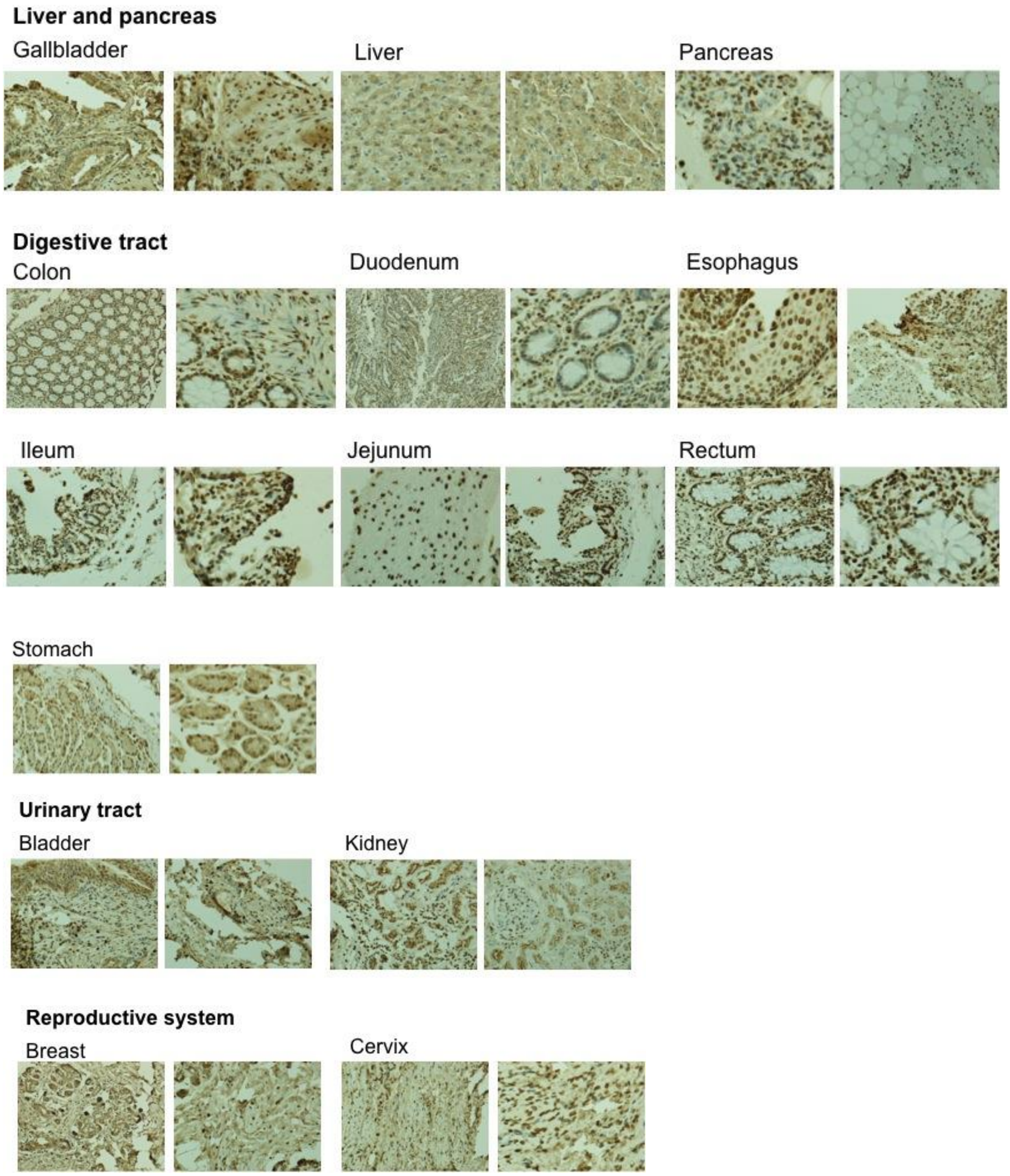

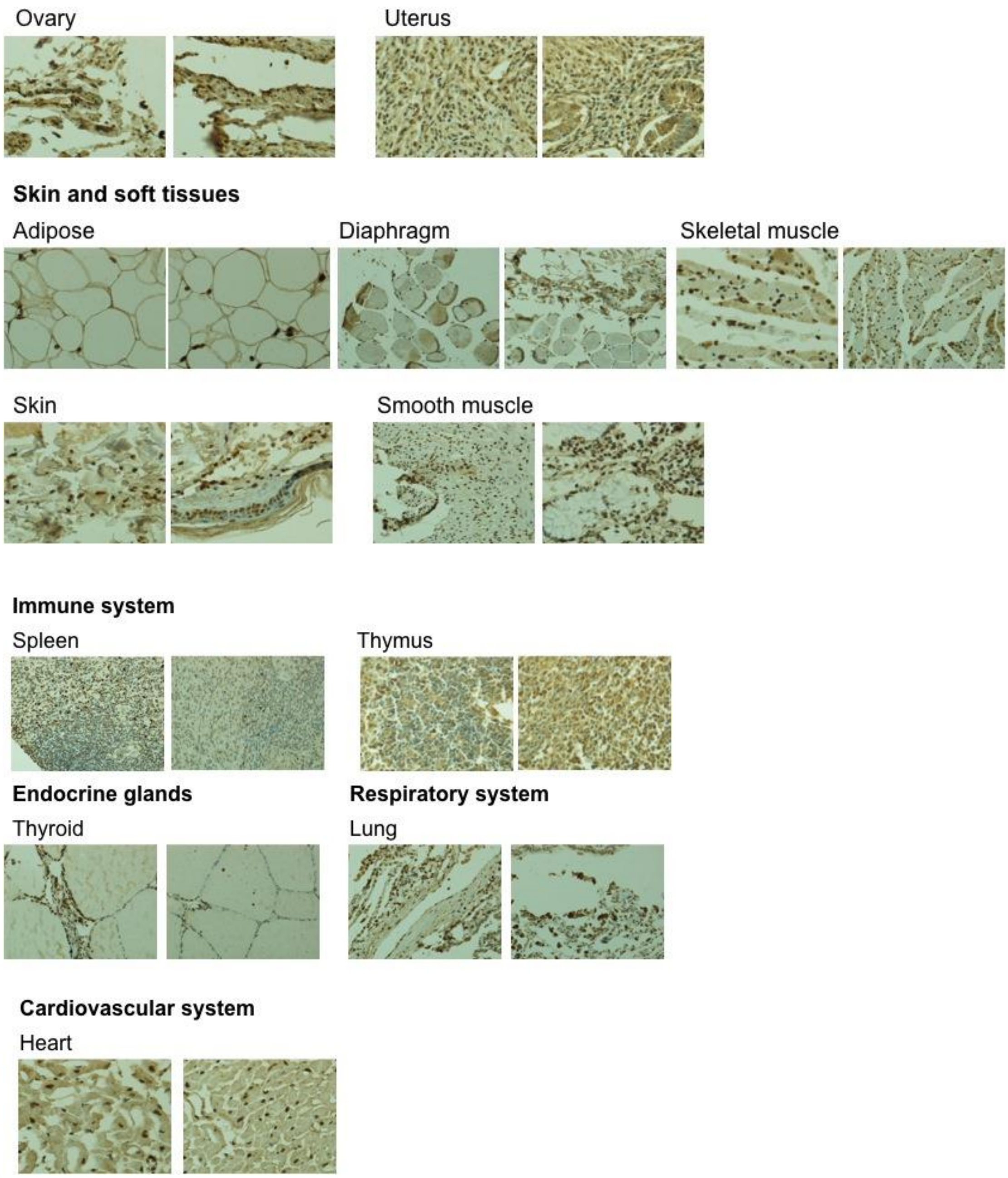
Ovary
Uterus
Skin and soft tissues
Adipose
Diaphragm
Skeletal muscle
Skin
Smooth muscle
Immune system
Spleen
Thymus
Endocrine glands
Thyroid
Respiratory system
Lung
Cardiovascular system
Heart

LLT1 is highly expressed in immune-privileged sites. Lectin-like transcript 1 (LLT1) and isotype control stainings of testes (A), brain (B) and placenta (C) using the anti-LLT1 2H7 mAb (1/500). 5x, 10x and 20x magnification (all stainings undertaken for Figure 4).

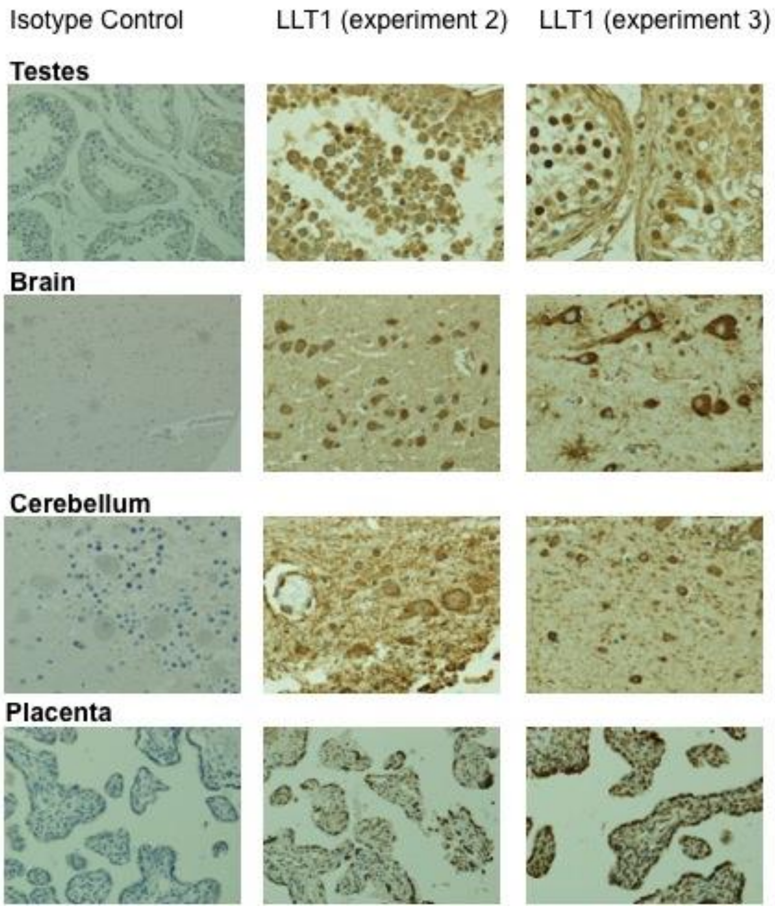

All FACS output for Figure 5. LLT1 inhibits NK cell degranulation. Expression of CD107a was measured by flow cytometry on NK cells incubated with 300.19- lectin-like transcript 1 (LLT1) as targets (green) or untransfected 300.19 cells (blue).

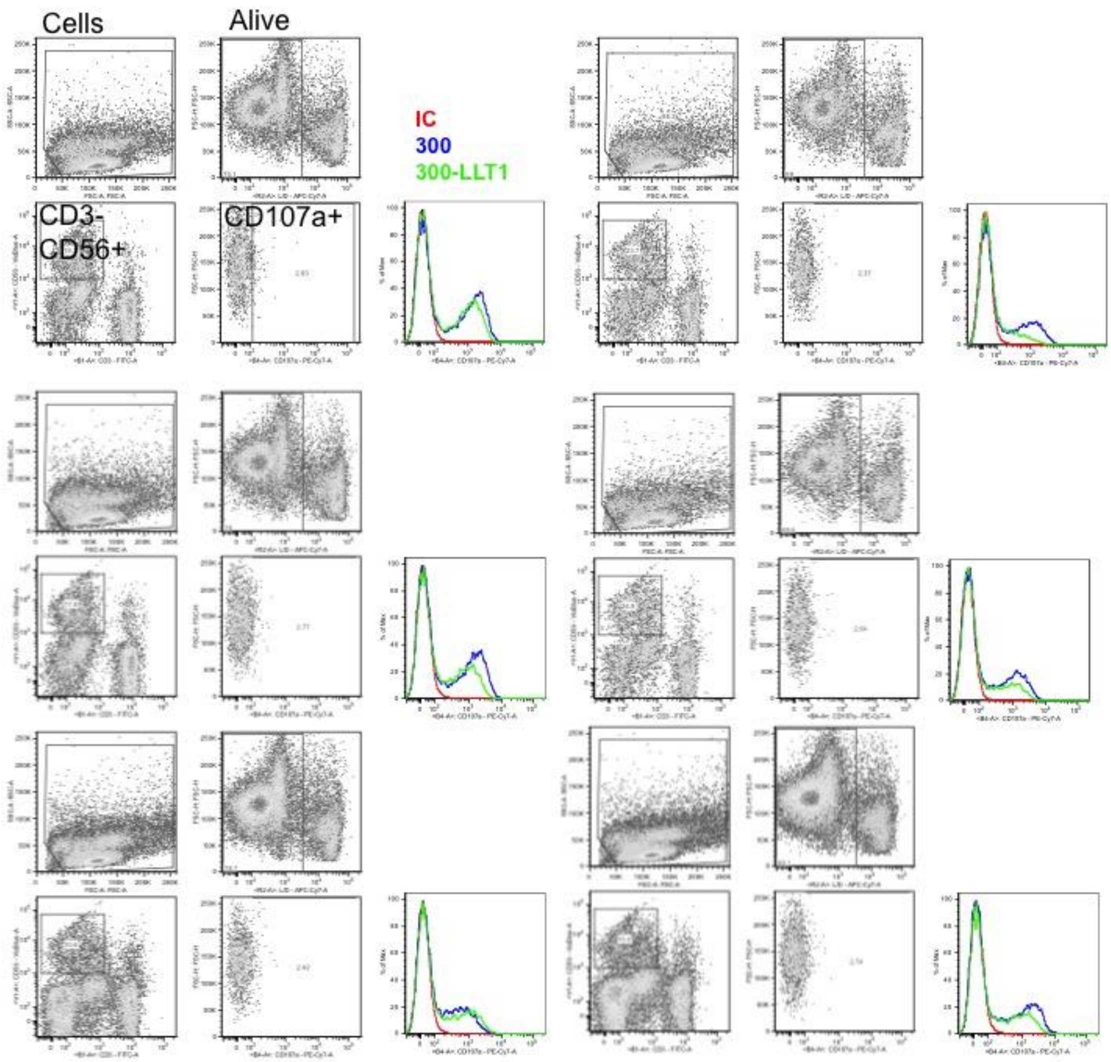

LLT1 staining of human tumor tissues. Staining of healthy and cancerous human tissues was performed using the 2H7 antibody (1/500). Changes in Lectin-Like Transcript 1 (LLT1) expression were tissue dependent. 5x, 10x and 20x magnification (all stainings undertaken for Supplementary Figure 1).

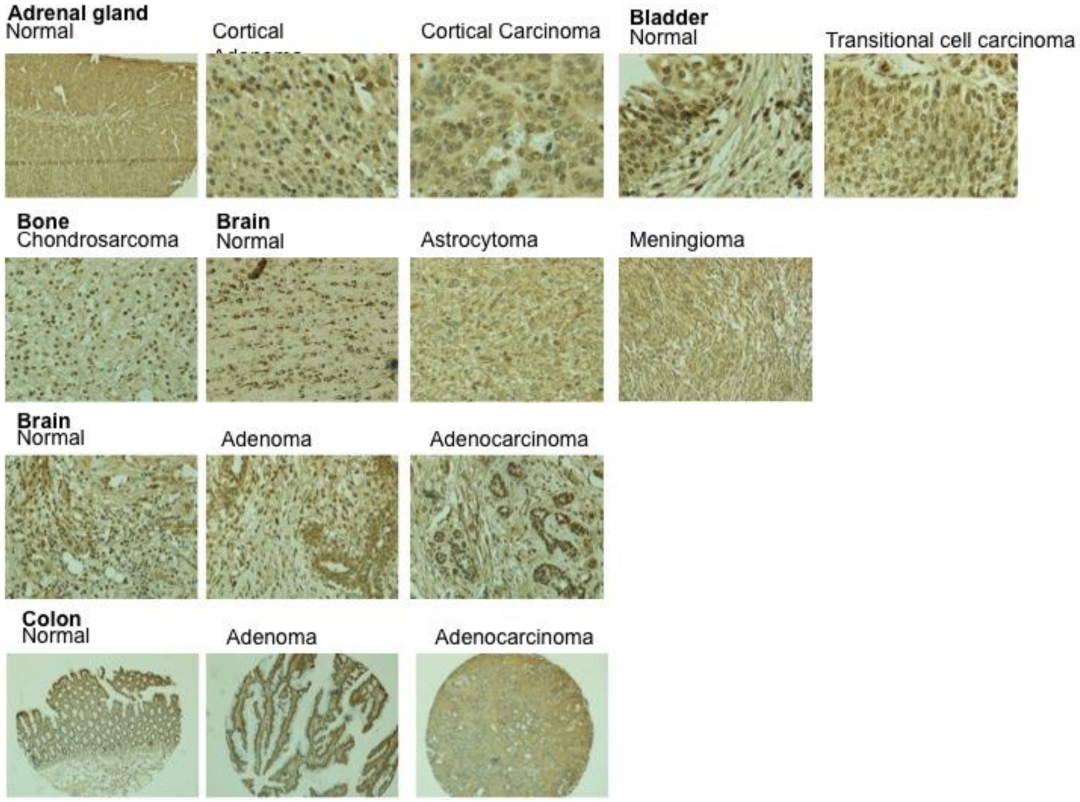

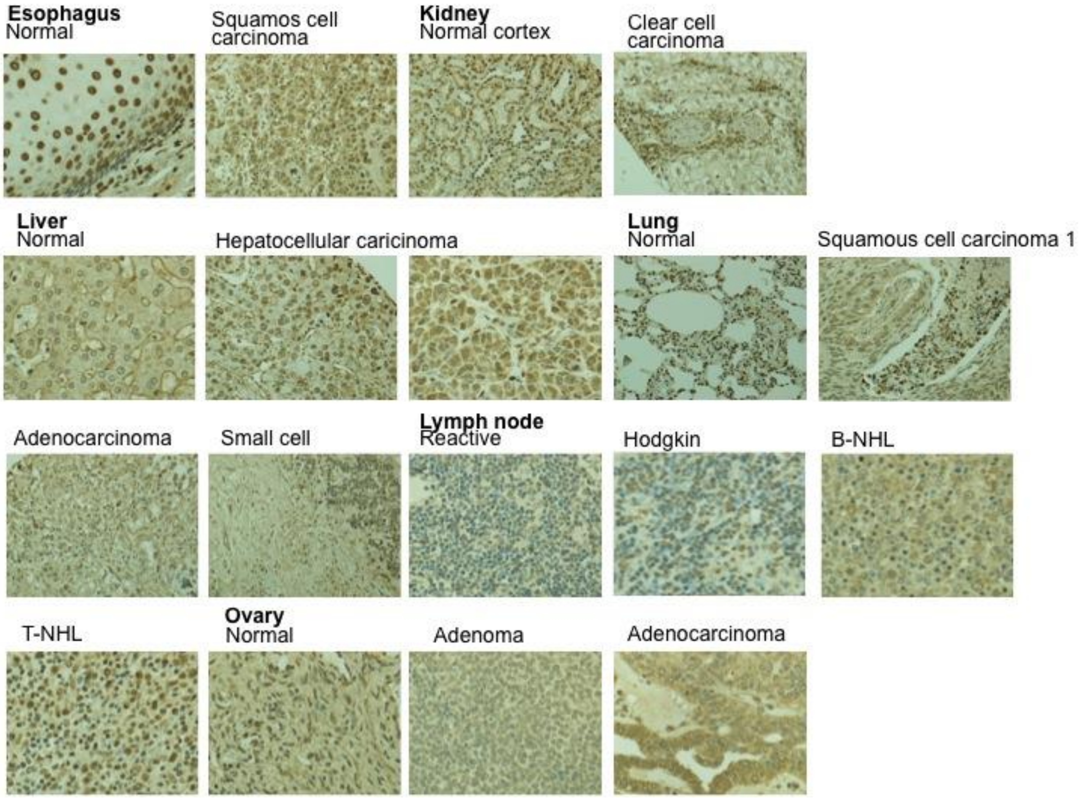
Esophagus
Normal
Squamos cell carcinoma
Kidney
Normal cortex
Clear cell carcinoma
Liver
Normal
Hepatocellular caricinoma
Lung
Normal
Squamous cell carcinoma 1
Adenocarcinoma
Small cell
Lymph node
Reactive
Hodgkin
B-NHL
T-NHL
Ovary
Normal
Adenoma
Adenocarcinoma

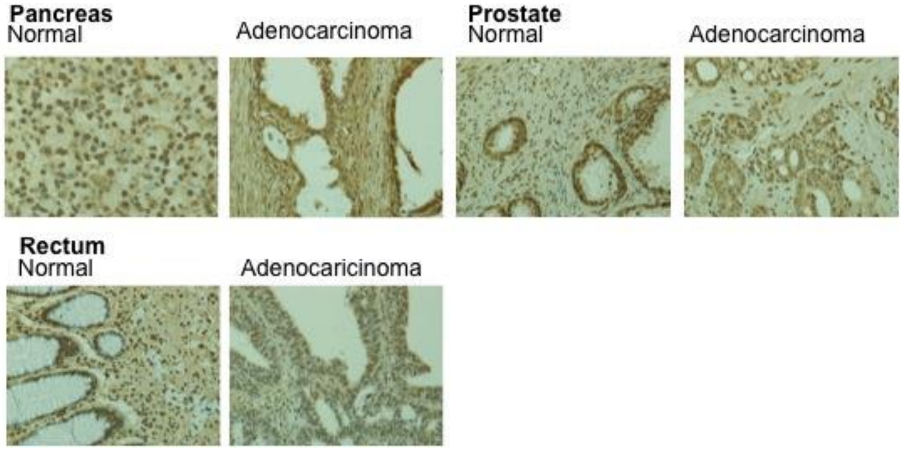
Pancreas
Normal
Adenocarcinoma
Prostate
Normal
Adenocarcinoma
Rectum
Normal
Adenocaricinoma
