# Supplementary figures and images for: Expression of lectin-like transcript-1 in human tissues
Source: F1000Res. 2016 Dec 29;5:2929. [Version 1] doi: 10.12688/f1000research.10009.1 (PMC5365220; doi:10.12688/f1000research.10009.1)

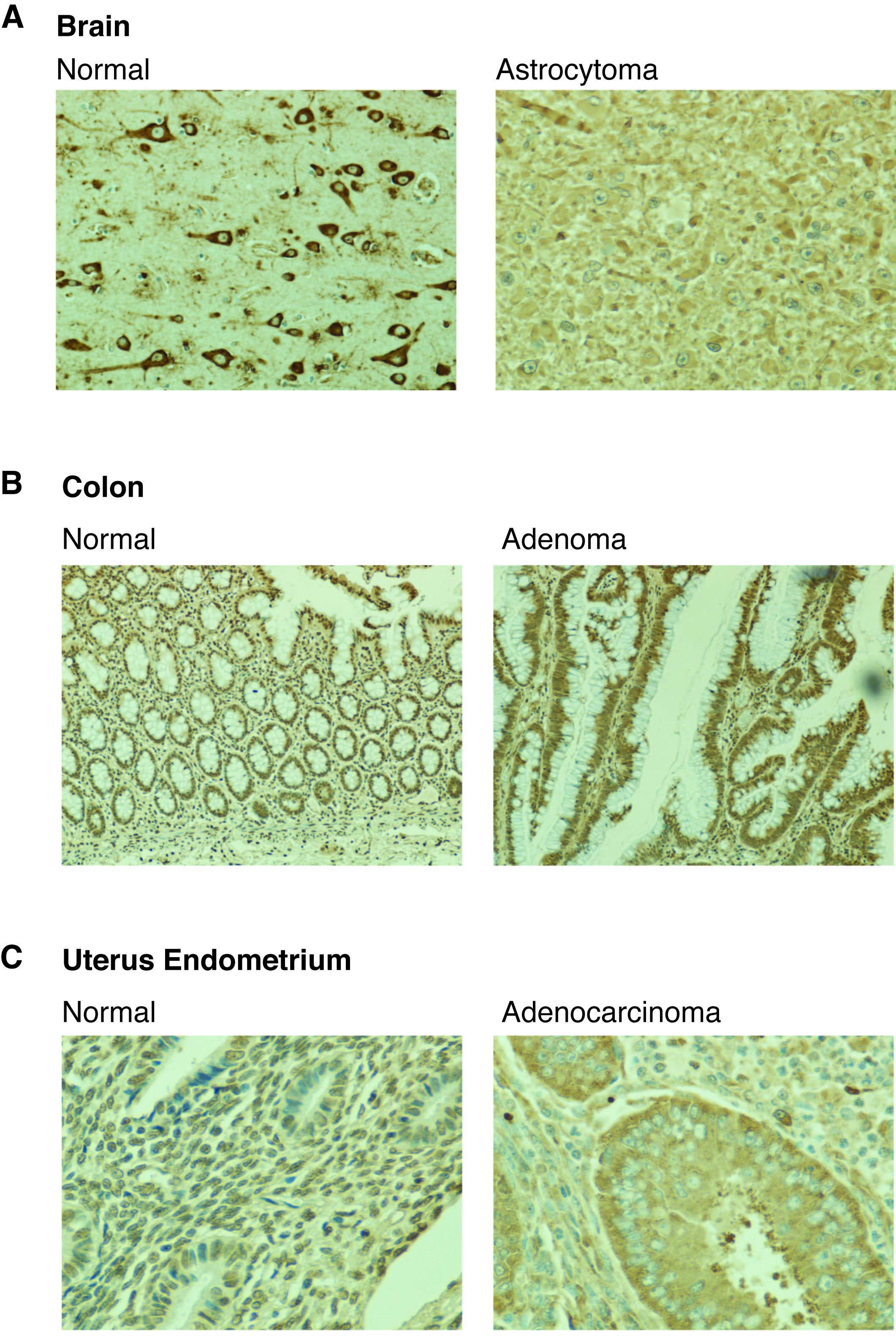

Supplement: Supplementary file 2 [file f1000research-5-10785-s0001.tgz › d52b6be9-1c7e-4e17-9a5c-931b4731b406.jpg]
