# Supplementary material for: Expression of lectin-like transcript-1 in human tissues
Source: F1000Res. 2016 Dec 29;5:2929. [Version 1] doi: 10.12688/f1000research.10009.1 (PMC5365220; doi:10.12688/f1000research.10009.1)
Supplement: Peer review history

# Peer review: Referee response for version 1

**DOI:** 10.5256/f1000research.10785.r20406

**Published:** 2017-03-20

**Authors:** Fiona Culley

**Affiliations:** 1 Respiratory Infections Section, National Heart and Lung Institute, Imperial College London, London, UK

The authors present data on the distribution of LLT1 in human tissues, obtained by flow cytometric analysis of PBMC and by immunohistochemical analysis of diverse tissues, using a novel monoclonal antibody 2H7. The current literature is inconstant in this regard, so this paper will be a useful reference point for those studying of the biology of LLT1.

 Can the authors provide more information on how the antibody and cell lines used to test it were originally generated and selected in the laboratory of Gordon Freeman, or provide a reference.

 In the results section describing Figure 1, the authors state that 2H7 is a good tool for studying distribution in tissue, but the methods section state that for Fig 1D, where immunofluorescence is shown, the antibody AF3480 is used. Is this correct? Can the authors clarify why a different antibody was used here?

 Please clarify what the 6 histograms in each part of Figure 2 represent. The columns are labelled 24h and 48h, but this does not appear to correspond to the figure legend.

 The authors should add scale bars to the images, rather than stating the magnification of the objective lens.

 Please correct IgG2A to IgG2a throughout.

 The authors should state the reference number for the ethical permission obtained.

I have read this submission. I believe that I have an appropriate level of expertise to confirm that it is of an acceptable scientific standard, however I have significant reservations, as outlined above.

# Peer review: Referee response for version 1

**DOI:** 10.5256/f1000research.10785.r20405

**Published:** 2017-03-08

**Authors:** Ondřej Vaněk, Jan Bláha

**Affiliations:** 1 Department of Biochemistry, Faculty of Science, Charles University, Prague, Czech Republic

The present study by A. Llibre *et al.* is describing novel monoclonal antibody, clone 2H7, that is able to detect protein LLT1 in various healthy as well as tumour human tissues, as shown by immunohistochemistry, fluorescence microscopy and flow cytometry. LLT1 was shown to have broad expression pattern with the highest expression levels detected in circulating B cells and monocytes and, surprisingly, also in immune-privileged sites - brain, placenta, testes. This observation supports its role in inhibition of NK cell cytotoxicity mediated by its interaction with inhibitory NK cell receptor NKR-P1. LLT1 inhibitory properties were confirmed in the present study using NK cell degranulation assay.

 Overall, this study is of general interest, mainly to the scientists studying directly this receptor:ligand interaction pair or other closely related pairs. Given the fact that LLT1 and especially NKR-P1 are linked to multiple human immune pathologies or other diseases, detailed characterization of LLT1 expression is adding valuable piece of information to this field.

 However, as already pointed out by Prof. Lanier in his peer review, I would also argue that for maximising impact of this work and its possible benefit to the scientific community, the availability of this novel antibody should be addressed, as well as the information whether it is blocking the LLT1:NKR-P1 interaction or not. Also, as already mentioned in preceding review, the use of blocking antibody (possibly 2H7) should have been included in degranulation assay, as it is a standard control setup in such experiments. This would also directly show blocking capabilities of 2H7 clone.

We have read this submission. We believe that we have an appropriate level of expertise to confirm that it is of an acceptable scientific standard.

# Peer review: Referee response for version 1

**DOI:** 10.5256/f1000research.10785.r18845

**Published:** 2017-01-03

**Authors:** Lewis Lanier

**Affiliations:** 1 Department of Microbiology and Immunology, University of California San Francisco, San Francisco, CA, USA

The authors present a comprehensive analysis of the LLT1 (CLEC2D) protein on human cells and tissues using a newly generated monoclonal antibody, clone 2H7, using flow cytometry and immunohistochemistry. In general, the information will be of interest to the scientific community studying this ligand - receptor (CD161) pair. The following points should be addressed:

1. When describing the amounts of antibody used to stain cells or tissues, the absolute concentration of the antibody should be stated (e.g. 1 ug/ml, etc.), rather than "1/500", which is meaningless unless the protein concentration of the antibody solution is provided. In the Materials mention a solution of 1.42 mg/ml, so is this then diluted 1/500? It would be clearer to simply state the concentration used in the staining experiment rather than "1/500".
2. The authors show decreased degranulation when NK cells were co-cultured with a cell line transfected with LLT1. However, subclones of cell lines or transfectants can vary in their ability to induce NK cell degranulation that is independent of the transduced gene or cDNA. The authors should provide a control experiment in which the LLT1-induced inhibition is reversed in the presence of a blocking antibody against CD161 and LLT1. Does the 2H7 antibody block the CD161-LLT1 interaction? This is important information that should be included in the revised paper.
3. The authors should comment on how surface expression of LLT1 relates to expression of LLT1 transcripts in these cell populations. Do they correlate or not?
4. PMA and ionomycin were used to stimulate the PBMC before analysis for LLT1 expression. It is well know that PMA can induce shedding of many cell surface proteins, for example CD4. Is LLT1 affected by PMA? This should be investigated and reported. 
5. Is the 2H7 antibody available commercially or will the authors deposit the hybridoma in the ATCC so it is available to others in the community? This is important so that other investigators can take advantage of this new information about LLT1 expression.

I have read this submission. I believe that I have an appropriate level of expertise to confirm that it is of an acceptable scientific standard, however I have significant reservations, as outlined above.
